# Theoretical Study of Fe^3+^ and Ni^2+^ Ion Interactions in Ethaline as the Deep Eutectic Solvent and Water Solutions Using Molecular Dynamics, Quantum Theory of Atoms in Molecules, and Non-Covalent Interactions

**DOI:** 10.1021/acsomega.4c08992

**Published:** 2025-04-16

**Authors:** Laudenor Amorim, Renato Veríssimo de Oliveira, Lucas Lima Bezerra, Pierre Basílio Almeida Fechine, Adriana Nunes Correia, Pedro de Lima-Neto, Norberto de Kássio Vieira Monteiro

**Affiliations:** † Department of Analytical Chemistry and Physical Chemistry, Science Center, Federal University of Ceará, Pici Campus, Block 940, 60440-900 Fortaleza, Ceará, Brazil; ‡ Department of Metallurgical and Materials Engineering, Technology Center, Federal University of Ceará, Pici Campus, Block 729, 60440-554 Fortaleza, Ceará, Brazil

## Abstract

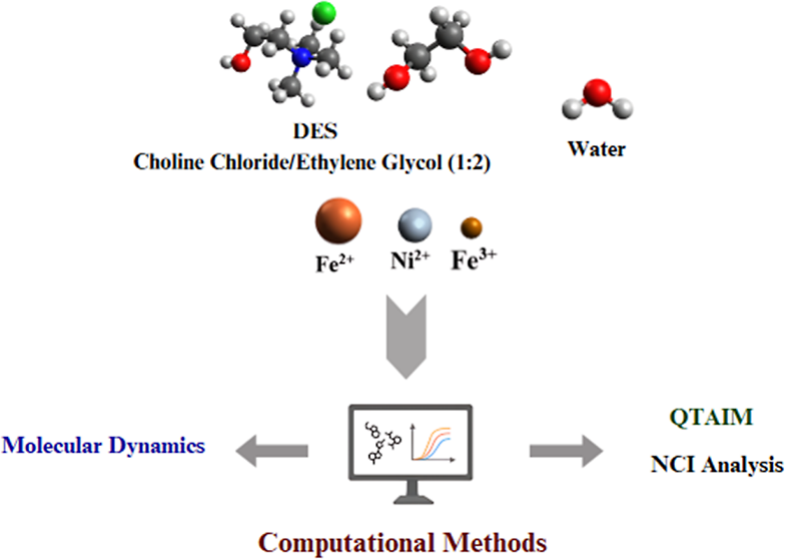

Deep eutectic solvents (DES) have several advantages compared to water and traditional solvents, making them an alternative, especially in applications that require better solvation, greater thermal stability, and a lower environmental impact. This study aimed to analyze the behavior of Fe^3+^ and Ni^2+^ ions in two quantities of water (300 and 5580 molecules) and in a eutectic solvent based on choline chloride and ethylene glycol (1ChCl:2EG). The computational methods used involved molecular dynamics, quantum theory of atoms in molecules (QTAIM), and noncovalent interactions simulations. Analysis of the radial distribution function multiplied by the number density [*g*(*r*)ρ] and the cumulative number (CN) indicated that the interactions between the metal ions and the water molecules were strongest for the systems with the most water. QTAIM determined the bond critical point, the electron density [ρ(*r*)], the Laplacian of the electronic density [∇^2^ρ(*r*)], and the electron localization function (η, ELF), allowing the interactions to be analyzed. Polarizability of the metal ions in both water and ethaline was compared; the increasing order of polarizability was Fe^3+^ < Ni^2+^ < Fe^2+^, with Fe^3+^ being the least polarizable due to its high charge and smaller ionic radius. In the mixed systems with Fe^2+^ or Fe^3+^ added to Ni^2+^, the metal species with the same charge competed similarly in water and DES. The intermolecular forces in DES are weaker due to the solvent’s lower polarity and dielectric constant than water. In the systems with the highest water content (5580 molecules), Fe^3+^ ions were surrounded by the largest water molecules, followed by Fe^2+^ and Ni^2+^. These results may help to better understand the solvation and behavior of these ions in different media, which has implications for use in electrodeposition, batteries, and corrosion inhibition.

## 1 Introduction

Deep eutectic solvents (DES) are formed from a mixture of Lewis or Bronsted acids and bases, creating a liquid mixture with a melting point lower than that of its pure components. They are usually formed from a hydrogen bond acceptor, which can be a metal complexed to a quaternary ammonium salt and a hydrogen bond donor such as alcohols, carboxylic acids, etc.^1−3^ The problems associated with using conventional solvents include flammability, toxicity, significant environmental impact, and high costs. DES also has adjustable physicochemical properties such as polarity, solubility, and viscosity. It has received widespread attention in various applications due to its advantages, such as high conductivity, wide electrochemical window, and environmental friendliness (low toxicity) compared to traditional solvents.^4,5^

DES can be used as reaction media for organic syntheses, such as protection/deprotection reactions, couplings, condensations, and cycloaddition, improving reaction rates and yields.^6^ Due to their ionic conductivity, they are also used in the electrodeposition of metals, batteries, and energy storage devices.^7^ The application of DES in the formation of protective layers can effectively reduce corrosion on metal surfaces, as in a specific composition of DES, composed of ascorbic acid and choline chloride, effectively preventing corrosion on copper surfaces. For copper-based materials, the potential of DES to prevent corrosion represents a significant advance, providing a more sustainable and effective approach.^8,9^ Due to its polarity and ability to form hydrogen bonds, water is one of the most widely used solvents. However, it has some limitations, such as the insolubility of nonpolar compounds such as oils and greases, small working temperature and pH range, and high surface tension. Therefore, the versatility of DES in several of these properties and their solubilization capacity make them useful compared to water limitations, for example.^10^

This study used ethaline as the DES (a 1:2 mol ratio mixture of choline chloride and ethylene glycol). Considering the possibilities, the research was aimed at the behavior of ions in solution (ion pairing) and ions with water (ion hydration), comparing the effects on the change from Fe^2+^ ion to Fe^3+^ in a mixture with the Ni^2+^ ion. In ionic hydration, the dissolved ions are surrounded by water molecules, with the cations attracted by the negative charges of the oxygen atoms and the anions by the positive charges of the hydrogen atoms, reducing the free energy of the system and favoring the stabilization of the ions in solution.^11^ In ion pairing, there are interactions between cations and anions in solution, forming ion pairs that can be loosely associated (solvent-separated ion pairs) or more closely associated (contact ion pairs). When the ions are close together, there is minimized interference from the solvent molecules, while in solvent-separated ion pairs the cation and anion are kept a certain distance apart, with one or more solvent molecules interposed between them. Although there is still an electrostatic attraction between the particles, the presence of the solvent reduces the intensity of this interaction, resulting in a weaker interaction, where the ions can move more independently. On the other hand, in contact ion pairs, with no solvent molecules between them, the cation and anion have a stronger electrostatic attraction and a more stable combination. This process can affect the solution’s electrical conductivity, the ions’ reactivity, and the stability of the ionic species in the solution.^12^

Computer models save time and reduce operating costs, making it possible to develop models that can help researchers study corrosion processes, electrodeposition, catalysis, etc. These models help with a detailed understanding of the underlying mechanisms, performing multiple simulations, and contributing to more sustainable research practices. The computational protocol adopted for the development of this work was based on previous publications by the author and collaborators of the research group,^13−17^ consisting of the use of classical molecular dynamics (MD) computer simulations,^18^ followed by quantum calculations using the quantum theory of atoms in molecules (QTAIM)^19^ and analysis of non-covalent interactions.^20−22^ Amorim et al. analyzed the behavior of Fe^2+^ and Mn^2+^ ions in isolation and an equimolar mixture in water and ethaline, keeping the temperature of 298 K constant.^15^ In this paper, the Mn^2+^ ion was replaced by Ni^2+^ (Fe–Ni systems) and Fe^2+^ by Fe^3+^ (Fe3–Ni systems) to observe the differences between the two species of iron ions.^23^

## 2 Computational Methods

### 2.1 Molecular Structures

The files with the structures of water, choline, and ethylene glycol were optimized using density functional theory (DFT) calculations using the Gaussian 09W software,^24^ employing the B3LYP functional hybrid in conjunction with the 6–311+G(d,p) basis set.^25^ The B3LYP/6–311+G(d,p) combination makes it possible to describe long-range interactions and regions of low electronic density by adding diffuse functions (+) on the heavy atoms and polarization functions (d,p) on all atoms.^26^ Using the Multiwfn 3.8 software, the partial charges were determined using the CHELPG method.^27^ This method was chosen for its ability to calculate partial atomic charges in molecules from electronic structure calculations, adjusting the charges to reproduce the molecular electrostatic potential in a grid of points around the molecule, which is fundamental for modeling the electronic structure and interactions, culminating in a more robust combination of methods. The study also used characterization techniques capable of identifying the intra- and intermolecular interactions between the Fe^2+^, Ni^2+^, and Fe^3+^ species alone and in a mixture in a 1:1 molar ratio for Fe^2+^ and Ni^2+^ ions and Fe^2+^ and Fe^3+^, both in the presence of water and ethaline. Thus, it was hoped to observe the behavior of the systems as well as to determine their chemical stability and physicochemical properties.

### 2.2 Molecular Dynamics Calculations

In the MD simulations, the initial configuration of the molecules was generated in GROMACS 2020.4^28^ using a cubic simulation box with dimensions of 8 nm × 8 nm × 8 nm, inserting the parameters into an input file (.mdp), using periodic boundary conditions to minimize edge effects in the system into which the molecules were inserted according to the systems detailed in Table 1. The quantities of each species in the systems were chosen to represent concentrations obtained experimentally.^13^ Two stages of energy minimization were then carried out with steepest descent algorithms,^29^ and the second was done with conjugate gradient^30^ minimization under the same convergence criteria. The convergence criterion adopted was 10.0 kJ × mol^–1^ × nm^–1^ s with the step size set at 0.0001 nm.

**Table 1:** Composition of the Systems Used^a^

systems		
FeCl_2_ with 1ChCl:2EG/FeDES	NiCl_2_ with 1ChCl:2EG/NiDES	FeCl_2_ and NiCl_2_ with 1ChCl:2EG/FeNiDES
ethylene glycol (800)	ethylene glycol (800)	ethylene glycol (800)
choline (400)	choline (400)	choline (400)
iron (9)	nickel (9)	iron (9)
chloride (418)	chloride (418)	nickel (9)
		chloride (436)

systems		
FeCl_2_ with H_2_O/FeW300	NiCl_2_ with H_2_O/NiW300	FeCl_2_ and NiCl_2_ with H_2_O/FeNiW300
iron (9)	nickel (9)	iron (9)
chloride (18)	chloride (18)	nickel (9)
water (300)	water (300)	chloride (36)
		water (300)

systems		
FeCl_2_ with H_2_O/FeW5580	NiCl_2_ with H_2_O/NiW5580	FeCl_2_ and NiCl_2_ with H_2_O/FeNiW5580
iron (9)	nickel (9)	iron (9)
chloride (18)	chloride (18)	nickel (9)
water (5580)	water (5580)	chloride (36)
		water (5580)

systems		
FeCl_3_ with H_2_O/Fe3W300	FeCl_3_ with H_2_O/Fe3W5580	FeCl_3_ and NiCl_2_ with H_2_O/Fe3NiW300
iron (9)	iron (9)	iron (9)
chloride (27)	chloride (27)	nickel (9)
water (300)	water (5580)	chloride (45)
		water (300)

systems		
FeCl_3_ and NiCl_2_ with H_2_O/Fe3NiW5580	FeCl_3_ with 1ChCl:2EG/Fe3DES	FeCl_3_ and NiCl_2_ with 1ChCl:2EG/Fe3NiDES
iron (9)	ethylene glycol (800)	ethylene glycol (800)
nickel (9)	choline (400)	choline (400)
chloride (45)	iron (9)	iron (9)
water (5580)	chloride (427)	nickel (9)
		chloride (445)

a FeDES, NiDES, and FeNiDES represent the Fe^2+^, Ni^2+^, and equimolar mixture (Fe^2+^ or Fe^3+^ with Ni^2+^) and Fe3DES, Fe3NiDES represent the Fe^3+^, Ni^2+^ systems in DES. FeW*X*, NiW*X*, FeNiW*X*, and Fe3NiW*X* represent the Fe^2+^, Ni^2+^, and Fe^3+^ in an equimolar mixture (Fe^2+^ or Fe^3+^ with Ni^2+^) systems in water, where *X*= 300 or 5580 water molecules added.

The particle mesh Ewald method was used for long-range electrostatic interactions, with cutoff radii of 1.0 nm for short-range and van der Waals interactions.^31^ Next, an *NVT* ensemble step was carried out, in which the box’s number of molecules, volume, temperature, and dimensions were fixed using the Berendsen thermostat and setting the temperature at 298 K.^32^ Then, the *NPT* ensemble stage was used, in which the pressure and temperature were fixed, allowing the volume of the simulation box to be dynamically adjusted employing the Parrinello–Rahman barostat,^33^ with an isotropic character, ensuring that the pressure of 1.0 bar is uniformly applied in all directions. The simulation time for the two ensembles was 20 ns. Finally, after the energy minimization and equilibrium steps, the MD simulation was carried out maintaining the previous temperature and pressure conditions and using the Leapfrog integrator to calculate the particle trajectories with the number of simulation steps set at 250,000,000 and with an integration step size of 2 fs, resulting in a total simulation time of 500 ns, which is adequate for the analysis of the systems studied.^34^

Effects of hydration and maintaining charge neutrality were evaluated, and the metal ions were simulated in ethaline and with two distinct quantities of water molecules: 300 and 5580. This approach was selected based on observations indicating negligible changes in other quantities within this range. The TIP3P model^35^ was used for the water molecules, and the Lennard-Jones parameters^36,37^ of the metal ions were inserted into the OPLS-AA force field topology file since it is widely used in simulations involving liquids in the literature and has been used in other publications by the authors.^38^

### 2.3 Single-Point Calculations, QTAIM, and NCI

This stage of the work followed the same computational detail as the authors’ previous studies.^15−17^ From the trajectory file (.xtc) generated in MD, a cut of 4.0 Å was made in the last frame of the simulations using visual molecular dynamics 1.9.4a53.^39^ It is worth noting that the spherical cuts using a maximum distance of 4 Å were performed precisely because they correspond to the inner sphere, based on the graphs of *g*(*r*)ρ and, given that the first solvation shell is located at a distance smaller than 4 Å, while the outer sphere is located above 4 Å.^40,41^ With the file generated, the Gaussian 09W software was used to calculate the single point using the M06-2X functional^42^ and the LANL2DZ basis set^43^ for the transition metals (Fe and Ni) and the 6–31+G(d, p) basis set for the other atoms present in the systems. QTAIM is fundamental for characterizing intra- and intermolecular interactions through the topology of electron density, describing how the flow of charge density is zero when distributed at any point on a molecular surface. According to QTAIM, the chemical structure is revealed by the stationary points called attractors and the electron density gradient paths ∇ρ that originate and end at these points, making it an alternative way of understanding the nature of chemical bonds.^44,45^ In covalent bonds, the electron density is concentrated between the atomic nuclei, forming a region of high density that stabilizes the interaction. For ionic bonds, QTAIM reveals a net transfer of electron density from one atom to another, resulting in partial charges, with maxima of ∇ρ being the nuclei of the system.^46^

With the aid of the Multiwfn 3.8^27^ software for QTAIM calculations, the results of the electronic structure, the density of all electrons ρ(*r*), the Laplacian of the electronic density ∇^2^ρ(*r*), and the electronic localization function were obtained, and the bond critical points (BCPs) were identified. The program also generated the files to plot the color map of noncovalent interactions (NCI) in Gnuplot 5.4.^47^ ELF was based on the electron density of the system, allowing a complete examination of the electron distribution in molecules and solids.^48^ This makes it easier to provide information on the characteristics of chemical bonds, chemical reactivity, structural stability, and the physical properties of the systems under investigation. Consequently, identifying areas characterized by high electron density (such as electron pairs in covalent bonds), areas of reduced density (such as atomic nuclei), and crucial zones, such as Bader’s critical points, becomes possible.^49^ These critical points serve as places where the electron density and its gradients are zero, thus playing a fundamental role in delineating intermolecular interactions and improving the understanding of the chemical attributes exhibited by the systems. NCI are chemical interactions between molecules that do not share or transfer electrons directly, including hydrogen bonds, ionic bonds, dipole–dipole interactions, and van der Waals forces. These forces impact the degree of solubility, reactivity, and stability of compounds. Although not as strong as covalent bonds, they form complexes, such as in the organization of proteins and nucleic acids and facilitate interactions between biomolecules.^50^ In addition, NCI play an important role in processes such as hydration and crystal formation.

Analyses of NCI are essential for visualizing attractive and repulsive interactions using reduced density gradient (RDG) graphs derived from the first derivative of the electronic density ρ (*s* = 1/(2(3π^2^)^1/3^)|∇ρ|/ρ^4/3^) versus sign(λ_2_)ρ, where λ_2_ is the second eigenvalue of the density’s Hessian matrix.^51^ The interpretation of these graphs is based on the analysis of the peaks, whose position on the *x*-axis indicates the type of interaction and the height on the *y*-axis correlates with the intensity of the interaction. In the region of sign(λ_2_)ρ < 0, the interactions are categorized as attractive, weak (sign(λ_2_)ρ ≈ 0), and repulsive (sign(λ_2_)ρ > 0). They are often represented by a color scheme (blue for attractive, red for repulsive, and green for weak interactions) as shown by the RDG isosurfaces mapped onto the molecule’s geometry. The regions to the left (sign(λ_2_)ρ < 0) have negative sign(λ_2_)ρ values, indicating interactions such as hydrogen bonds and π–π interactions. At the other end, positive values of (sign(λ_2_)ρ > 0) indicate repulsive interactions such as overlapping electron clouds. Regions near zero on the *x*-axis generally correspond to van der Waals interactions or regions with no significant interactions. In regions far from the molecular center, where the electron density is low and its gradient is relatively high, the RDG (>0.5) shows high values, which strongly indicates regions of weak or nonexistent interactions. However, in the vicinity of chemical bonds and zones of intermolecular interactions, where the electron density is significant, and its gradient tends toward small variations, the RDG approaches zero, allowing covalent bonds, NCI and other relevant characteristics of the molecular electron distribution to be visualized and quantified.^52−55^

NCI can be studied quantitatively by visual analysis of the RDG in the range 0.0–2.0, while for (sign λ_2_)ρ, it was −0.035 to 0.02 au This approach integrates the molecular real-space functions within the RDG surface, using a cutoff value of RDG = 0.5 to delimit the isosurface. RDG analysis makes it possible to identify and characterize specific regions where attractions and repulsions occur, contributing to understanding the stability and properties of the systems studied. By defining this cutoff value for RDG, the isosurface is delimited to grid points smaller than 0.5, meaning that only regions of molecular space with RDG values below 0.5 will be considered in the analysis. This choice is widely used to focus on the most significant interactions and exclude regions far from the molecule where the RDG tends to take on high values. In general, NCI is configured in regions with low values of RDG and the sign of the second eigenvalue of the electronic density Hessian matrix (sign(λ_2_)ρ).

## 3 Results and Discussion

**Figure 1:**
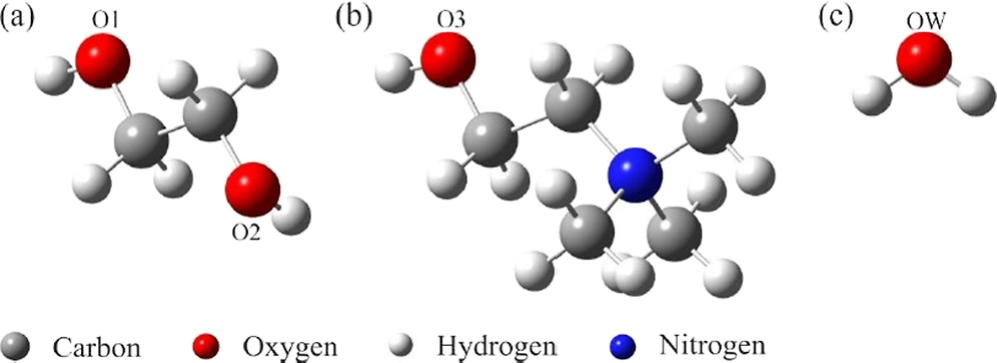
Structures used in the computational simulations and nomenclature of main atoms. (a) Ethylene glycol, where O1 and O2 represent their oxygen atoms; (b) choline, where O3 represents its oxygen atom; and (c) water, where OW represents the oxygen atom.

In this section, the information for the Fe^2+^ ion will be limited since the authors have already discussed this approach.^15^ The structures of the molecules used in the computer simulations can be seen in Figure 1, and the tables with the topological data obtained by QTAIM of BCPs for all systems are in the Supporting Information.

### 3.1 Validation of MD Simulations

Bezerra et al.^17^ validated the ethylene glycol, choline, and water parameters that were also used in this work, by correlating specific mass data obtained using the experimental and computational methods. An excellent correlation between these methods was observed, indicating that these parameters may be used to describe the ethylene glycol, choline, and water species.

### 3.2 Fe^3+^ in DES and Water (300 and 5580)

#### 3.2.1 Fe3DES

**Figure 2:**
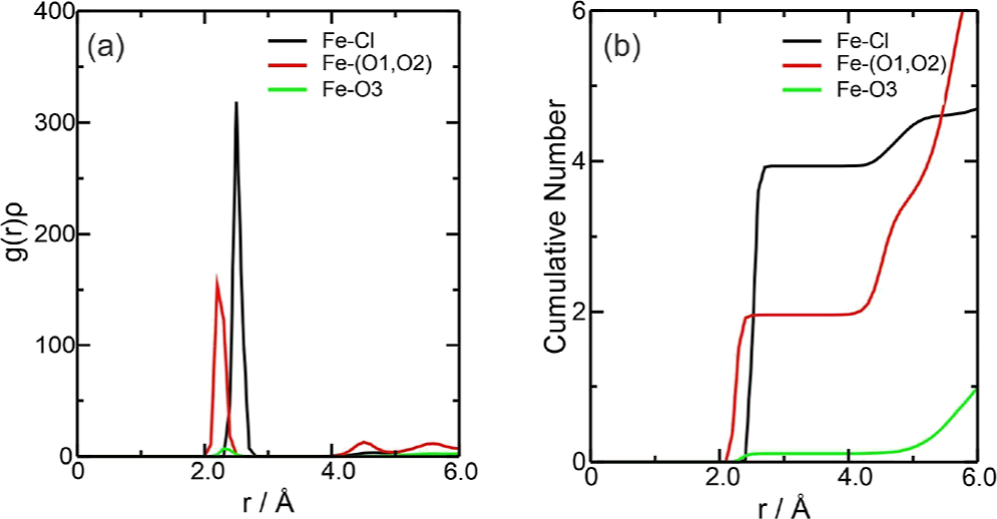
Radial distribution function multiplied by the average number density of the observed atoms, *g*(*r*)ρ, around the Fe^3+^ ions present in the (a) Fe3DES system and (b) CN of the counterions for this system. The color references in this figure are [(Fe3–Cl, black line), (Fe3–(O1, O2), red line), and (Fe3–O3, green line)].

The results for the Fe^2+^ ion have already been discussed in another article and will therefore not be discussed again. Figure 2a describes the density probability of the system components around the Fe^3+^ ion, represented by the radial distribution function [*g*(*r*) or RDF] multiplied by the average number density (ρ) of the observed atoms [*g*(*r*)ρ].^56,57^Figure 2b shows the cumulative number (CN) of the species around the Fe^3+^ center, derived from integrating the radial pair distribution function, *g*(*r*).^58^ CN analysis helps to understand the number of neighboring molecules in the first solvation layer. In DES, as the Fe charge increased, the *g*(*r*)ρ graph showed that the main interaction was between the Fe^3+^ ion and Cl^–^ at around 2.46 Å, followed by the interaction with ethylene glycol at around 2.20 Å at the start of the interaction (Figure 2a). Comparing the Fe–Cl interaction with the iron ions, it can be seen that the Fe–Cl interactions for the Fe^3+^ ion are stronger than those for the Fe^2+^ ion; however, the interaction between Fe–(O1, O2) was stronger for Fe^2+^. The results suggest that the Fe^3+^ ion has a [Ar] 3d^5^ electron configuration, with the 3d orbitals filled, which results in a less stable orbital overlap between the iron and the water molecules, weakening the metal–ligand bond compared to the Fe^2+^ complex, which has an [Ar] 3d^6^ configuration.^59^ The interaction of choline with oxygen was insignificant, following the same trend as that of the Fe^2+^ ion. According to the CN in Figure 2b, approximately 4.0 chloride ions and 2.0 ethylene glycol molecules complex Fe^3+^. For the Fe3–Cl interaction, there are values of Σρ(*r*) = 1.447 × 10^–1^ and Ση(*r*) = 4.743 × 10^–1^ while for the Fe3–(O1, O2) interaction, Σρ(*r*) = 5.880 × 10^–2^ and Ση(*r*) = 5.285 × 10^–2^, showing that the effect of increasing the charge of iron implied modifications to its electron cloud (Figure 4).

**Figure 3:**
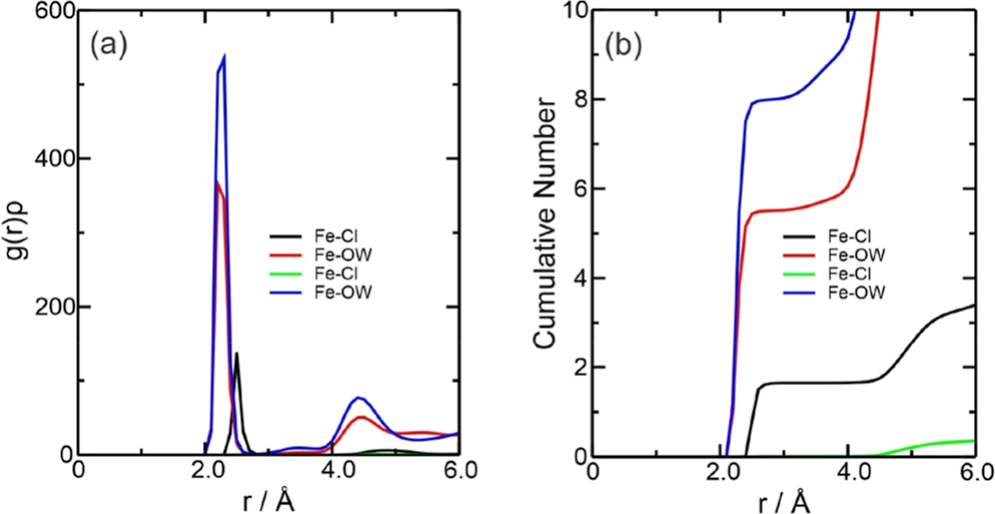
Radial distribution function multiplied by the average number density of the observed atoms, *g*(*r*)ρ, around the Fe^3+^ ions present in the (a) Fe3W*X* systems (where *X* = 300 or 5580 water molecules added) and (b) CN of the counterions for these systems. The color references in this figure are [water 300, (Fe3–Cl, black line), (Fe3-OW, red line) and water 5580, (Fe3–Cl, green line), and (Fe3-OW, blue line)].

**Figure 4:**
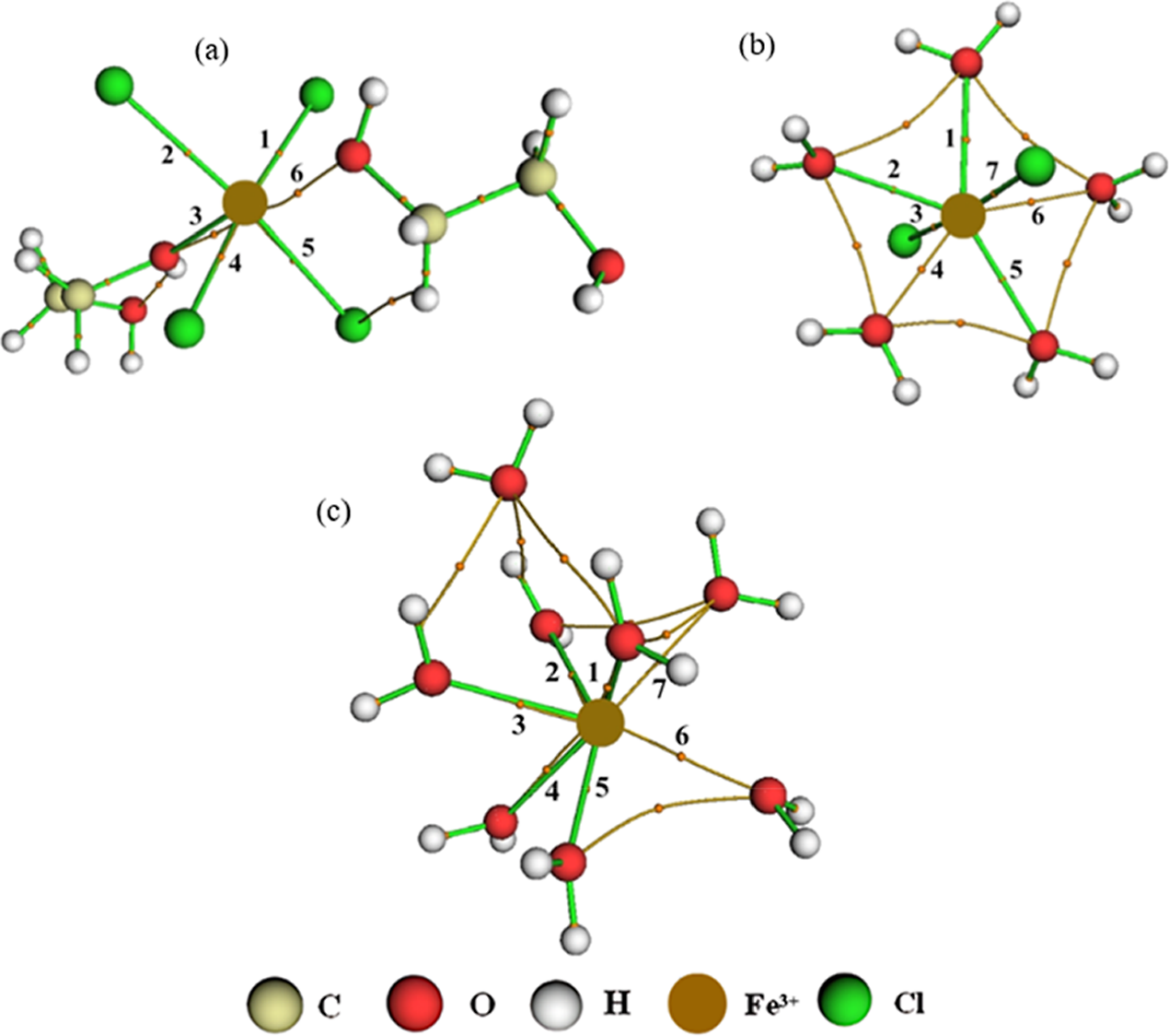
BCPs and intermolecular interactions for Fe^3+^ ion at the system (a) Fe3DES, (b) Fe3W300, and (c) Fe3W5580.

#### 3.2.2 Fe3W300 and Fe3W5580

The distinction between ion hydration and ion pairing lies in the interactions involved: hydration involves the interaction of ions with water molecules, while pairing involves the association of oppositely charged ions. Fe^3+^ has a smaller ionic radius than Fe^2+^, presenting a greater charge density and forming stronger bonds with ligands such as H_2_O, OH^–^, and F^–^, making it less reactive and more intense hydration than the Fe^2+^ ion. However, this difference in size is relatively small, and other factors, such as the electron configuration and the nature of the ligand, also play an important role in the stability and reactivity of the complexes formed.^60^

The graphs of the *g*(*r*)ρ function showed that for both amounts of water, although both Fe^2+^ and Fe^3+^ have well-defined Fe-OW peaks in *g*(*r*)ρ—showing clear coordination layers—Fe^3+^ tends to have sharper peaks, smaller Fe–Cl and Fe-OW distances, and greater stability in its hydration sphere due to its greater charge (Figure 3a).^61,62^ The peaks for the first interaction with water overlapped in both quantities, occurring around 2.27 Å, and the second peak was at 4.40 Å. The interaction with the Cl^–^ ion was not detected for W5580. The CN (Figure 3b) showed around 1.69 Cl^–^ ions around Fe^3+^ and 5.49 and 7.95 water molecules for W300 and W5580, respectively. For the Fe3W300 system, the Fe–Cl interaction showed values of Σρ(*r*) = 8.396 × 10^–2^ and Ση(*r*) = 1.520 × 10^–1^, followed by the Fe-OW interaction with Σρ(*r*) = 1.791 × 10^–1^ and Ση(*r*) = 6.891 × 10^–1^. In Fe3W5580, Fe–Cl showed no values while Fe-OW had Σρ(*r*) = 2.702 × 10^–1^ and Ση(*r*) = 1.201, showing the strong presence of water around the Fe^3+^ ion (Figure 4b,c).

### 3.3 Ni in DES and Water (300 and 5580)

#### 3.3.1 NiDES

**Figure 5:**
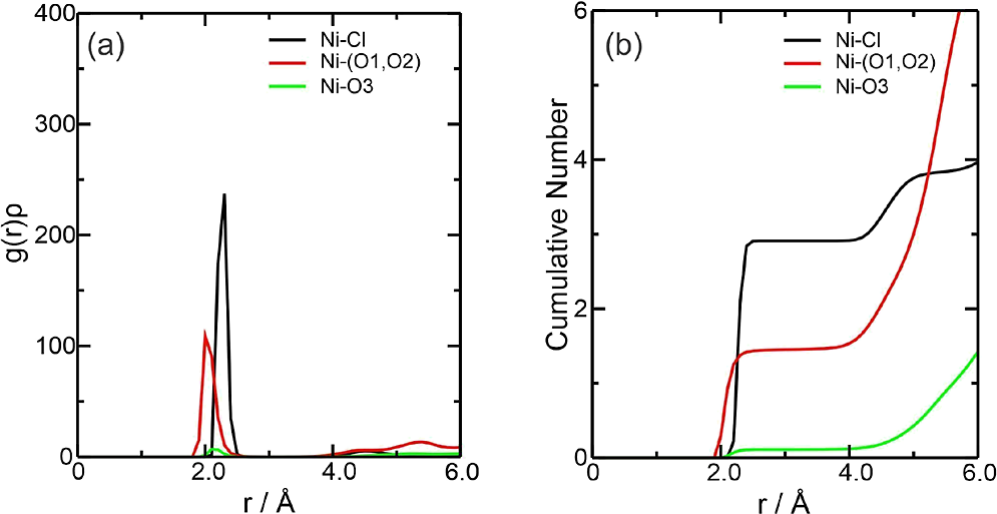
Radial distribution function multiplied by the average number density of the observed atoms, *g*(*r*)ρ, around the Ni^2+^ ions present in the (a) NiDES system and (b) CN of the counterions for this system. The color references in this figure are [(Ni–Cl, black line), (Ni–(O1, O2), red line), and (Ni–O3, green line)].

In DES, the graph of *g*(*r*)ρ showed that the main interaction was between the Ni^2+^ ion with Cl^–^ at around 2.2 Å, followed by the interaction with ethylene glycol at around 2.0 Å at the start of the interaction (Figure 5a). The behavior of these two interactions for Ni^2+^ is similar to that of Fe^2+^ due to the strong electrostatic attraction between Ni^2+^ and Cl^–^ ions. However, the Ni–Cl interaction proved to be slightly stronger than the Fe–Cl interactions for the Fe^2+^ and Fe^3+^ ions. Still, the interaction with the oxygen of the choline (Ni–O3) was insignificant, in the same way as for the other species. According to Figure 5b, there are, on average, approximately 3 chloride ions and 1.4 ethylene glycol molecules complexing Ni^2+^. For the Ni–Cl interaction, there are values of Σρ(*r*) = 1.875 × 10^–1^ and Ση(*r*) = 3.907 × 10^–1^, while for the Ni–(O1, O2) interaction, surprisingly no values appeared, as can be seen by the absence of BCPs in the electron density for this interaction (Figure 9a), which is an indication that they are not strong enough to be detected by QTAIM (See Supporting Information).

**Figure 6:**
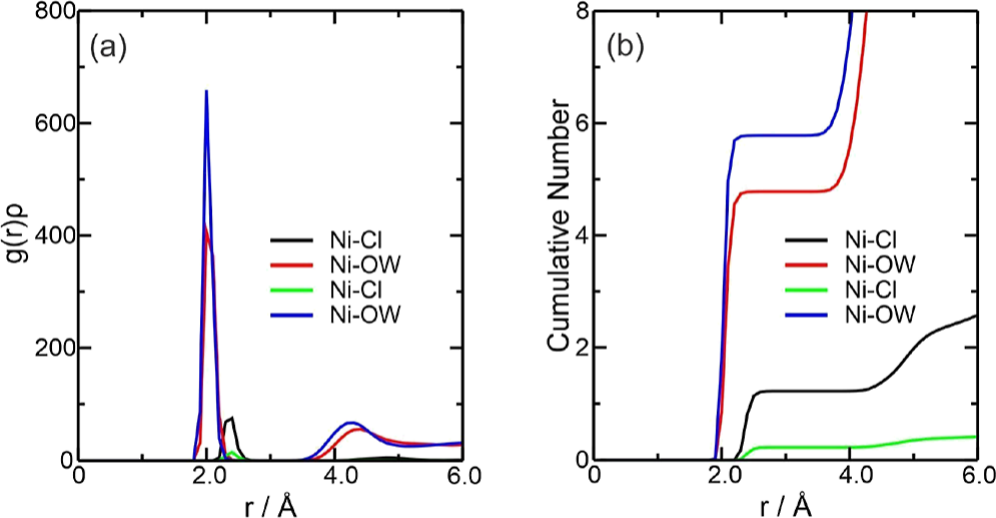
Radial distribution function multiplied by the average number density of the observed atoms, *g*(*r*)ρ, around the Ni^2+^ ions present in the (a) NiW*X* systems (where *X* = 300 or 5580 water molecules added) and (b) CN of the counterions for these systems. The color references in this figure are [water 300, (Ni–Cl, black line), (Ni-OW, red line) and water 5580, (Ni–Cl, green line), and (Ni-OW, blue line)].

**Figure 7:**
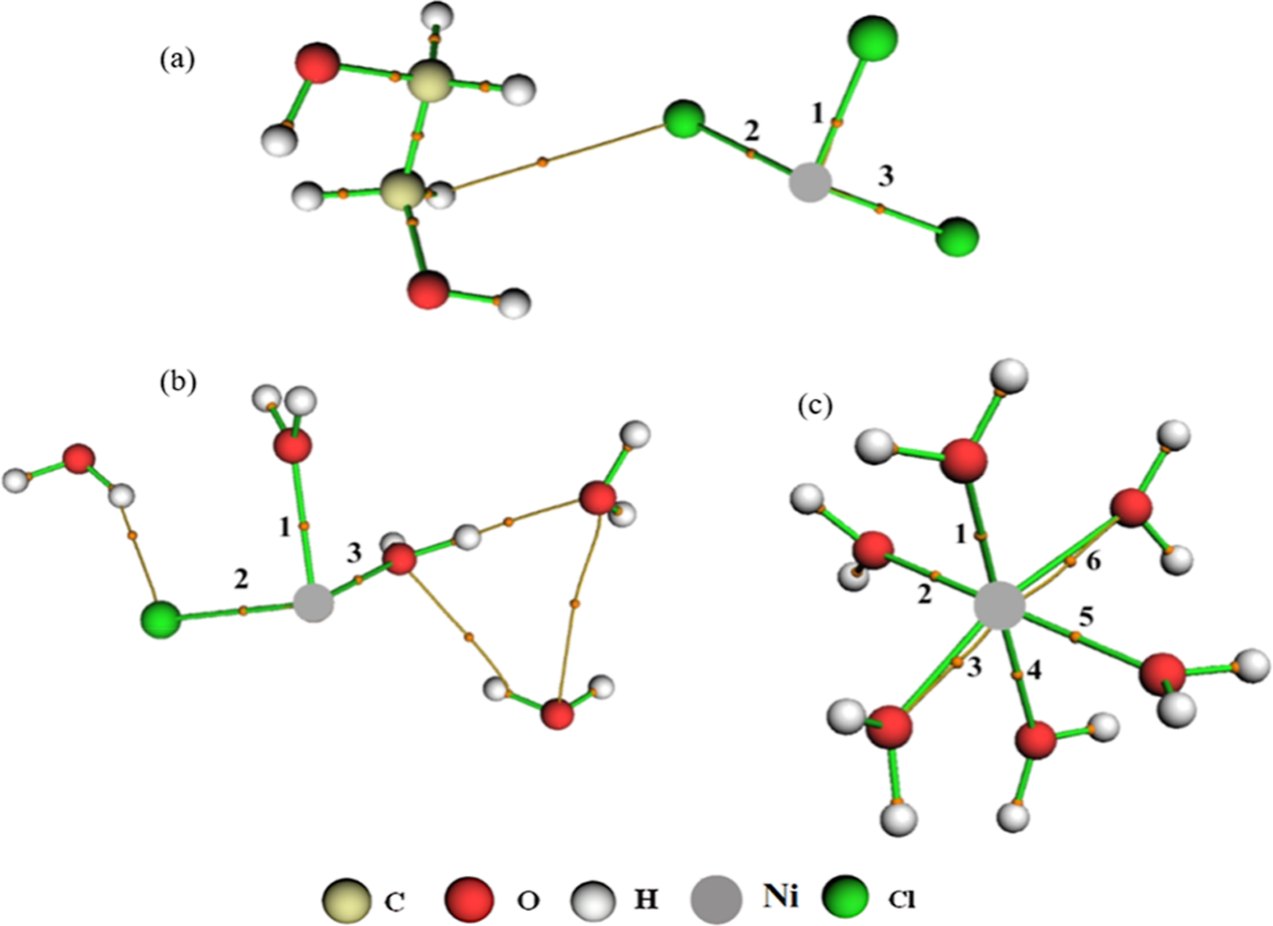
BCPs and intermolecular interactions for Ni^2+^ ion in the systems (a) NiDES, (b) NiW300, and (c) NiW5580.

**Figure 8:**
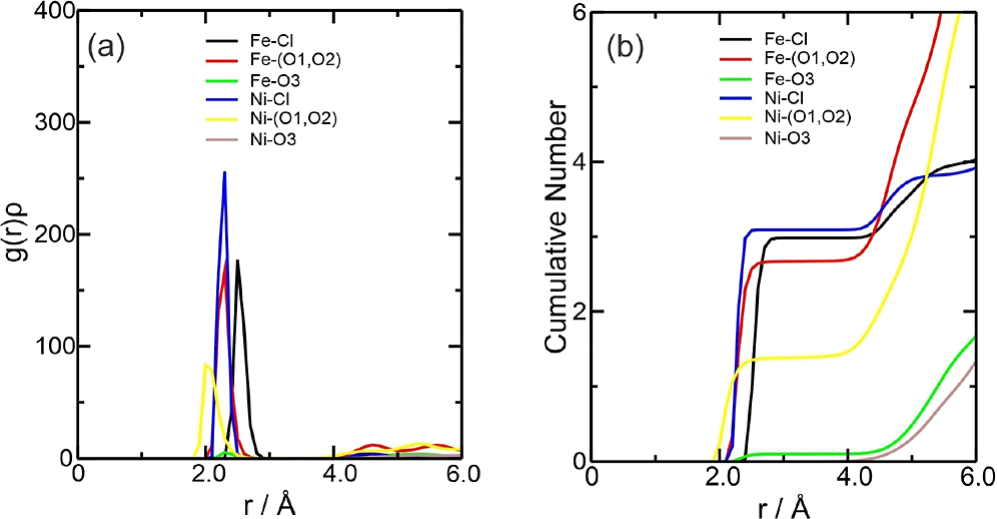
Radial distribution function multiplied by the average number density of the observed atoms, *g*(*r*)ρ, around the Fe^2+^ and Ni^2+^ ions present in the equimolar mixture (a) FeNiDES system and (b) CN of the counterions for this system. The color references in this figure are [(Fe–Cl, black line), (Fe–(O1, O2), red line), and (Fe–O3, green line)] and [(Ni–Cl, blue line), (Ni–(O1, O2), yellow line), and (Ni–O3, brown line)].

**Figure 9:**
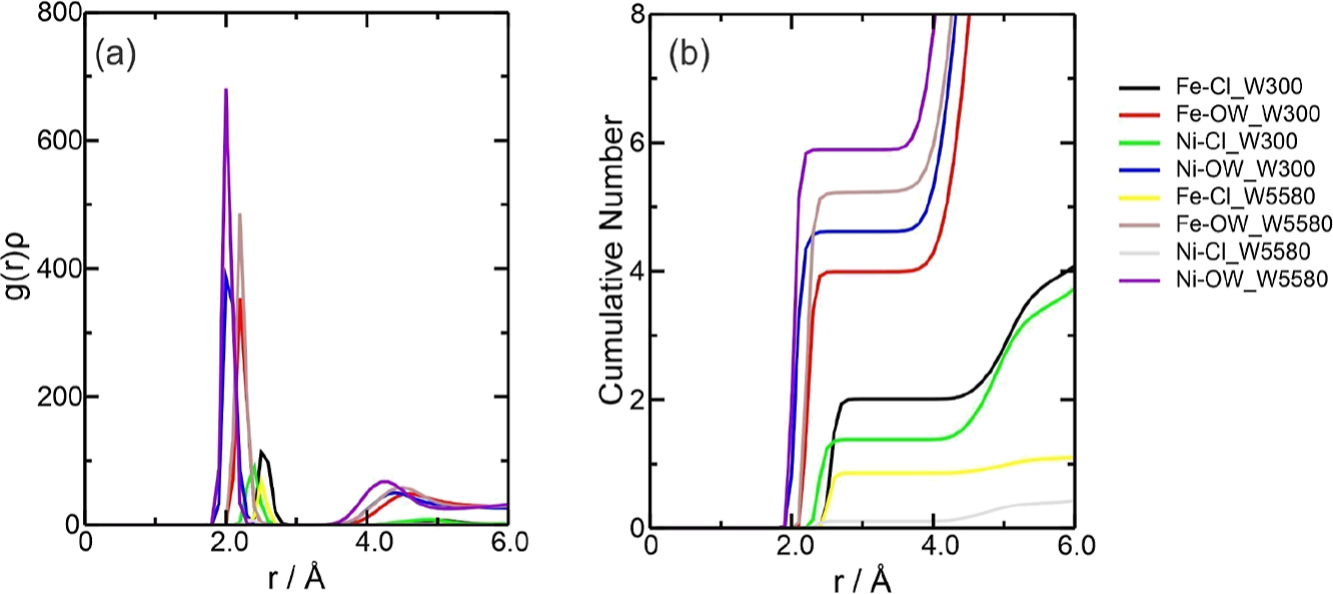
Radial distribution function multiplied by the average number density of the observed atoms, *g*(*r*)ρ, around the Fe^2+^ and Ni^2+^ ions present in the equimolar mixture (a) FeNiW*X* systems (where *X* = 300 or 5580 water molecules added) and (b) CN of the counterions for these systems. The color references in this figure are [water 300, (Fe–Cl, black line), (Fe-OW, red line), (Ni–Cl, green line), (Ni-OW, blue line) and water 5580, (Fe–Cl, yellow line), (Fe-OW, brown line), (Ni–Cl, gray line), and (Ni-OW, violet line)].

#### 3.3.2 NiW300 and NiW5580

For the Ni^2+^ species isolated in water, the behavior between the metal ion and water was similar, with two solvation layers observed and the peaks for the Ni-OW interaction overlapping at around 2.0 Å. The *g*(*r*)ρ results for the NiWater system (Figure 6a) indicated that the main interaction was between the Ni^2+^ ion and the oxygen in water (Ni-OW) around 2.0–2.2 Å, confirmed by the CN, with 4.79 water molecules for NiW300 and 5.81 for NiW5580 (Figure 6b). Therefore, this difference indicates that there is, on average, one more water molecule around Ni^2+^ in the NiW5580 system and, as we can see in Figure 7b,c of molecular graphs, the NiW5580 system contains one more water molecule than the NiW300 system. Furthermore, it is noted that the NiW300 system has 5 water molecules in its vicinity (4.79). The NiW5580 system has 6 molecules in its vicinity (5.81), thus showing an excellent correlation between the CN and the molecular graph (Figure 6b). A second Ni-OW solvation layer exists around 4.3 Å, which strongly indicates that Ni^2+^ coordination is octahedral in water. For the Ni–Cl interaction, the sum of all ρ(*r*) and η(*r*) values were NiW300, Σρ(*r*) = 5.512 × 10^–2^ and Ση(*r*) = 2.681 × 10^–1^; in NiW5580, no Ni–Cl interaction was detected. Considering the Ni-OW interaction, the values were NiW300, Σρ(*r*) = 1.215 × 10^–1^ and Ση(*r*) = 1.178 × 10^–1^; NiW5580, Σρ(*r*) = 3.551 × 10^–1^ and Ση(*r*) = 3.379 × 10^–1^ (Figure 7b,c).

### 3.4 FeNiDES and FeNiWater (300 and 5580)

#### 3.4.1 FeNiDES

**Figure 10:**
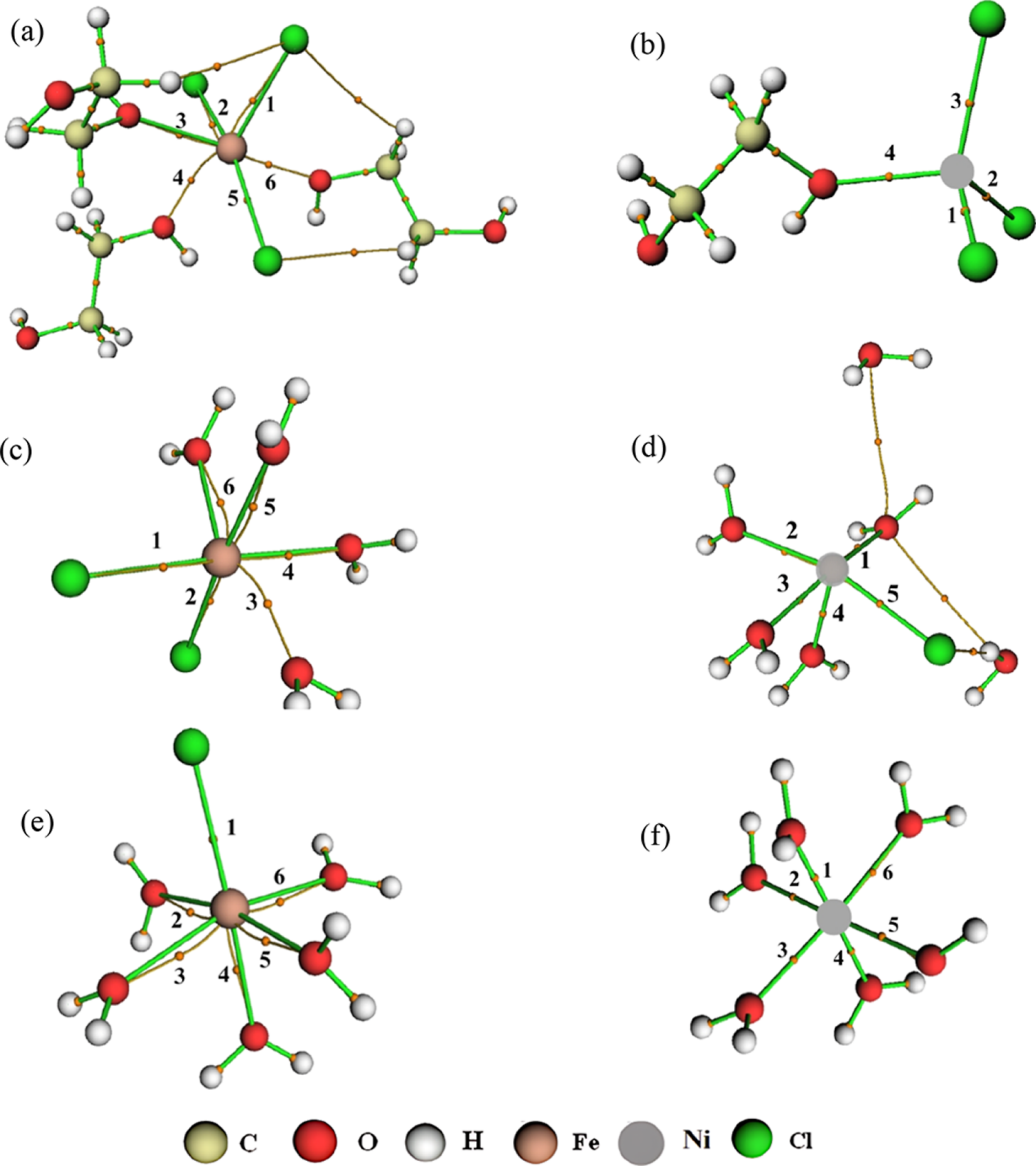
BCPs and intermolecular interactions for Fe^2+^ (a) and Ni^2+^ (b) ions with the FeNiDES systems; Fe^2+^ (c) and Ni^2+^ (d) ions with the FeNiW300 systems; Fe^2+^ (e) and Ni^2+^ (f) ions with the FeNiW5580 systems.

The size of the metal ion is a factor that influences the preference for ligands in the formation of complexes so that smaller ions tend to form more stable complexes with smaller ligands; larger ions prefer larger ligands, which is justified by the ion’s charge density and the ligands’ spatial accommodation. However, the difference in the ionic radii values of the Fe^2+^ and Ni^2+^ species is small, and the results for the equimolar mixture of these two species differed little from their systems.^63^ The *g*(*r*)ρ results for the FeNiDES system showed that the most significant interactions were between the metal ions and the Cl^–^ anion at around 2.28 Å for Ni–Cl and 2.46 Å for Fe–Cl. The Ni–(O1, O2) interactions were around 2.01 Å and Fe–(O1, O2) interactions were around 2.28 Å (Figure 8a), and the presence of two solvation layers was noted. According to Figure 10b, there are, on average, approximately 3.06 Cl^–^ ions and 2.70 ethylene glycol molecules complexing Fe^2+^ and 3.18 Cl^–^ ions and 1.36 ethylene glycol molecules complexing Ni^2+^, which suggests that mixing the metal ions caused small changes in the interactions with Cl^–^, demonstrating the competition between the ions to form more stable complexes.

Comparing these *g*(*r*)ρ and CN results with the FeDES and NiDES systems, the probability of Ni–Cl interaction is higher than for Fe–Cl, which the nephelauxetic effect can explain.^64,65^ In other words, it is a metal ion’s electron cloud expanding due to its interaction with a ligand. Thus, the smaller ionic radius of Ni^2+^ implies a higher charge density, resulting in stronger electrostatic interactions with Cl^–^. Another factor is due to the d^8^ electron configuration of Ni^2+^, which may contribute to a stronger bond with Cl^–^ compared to the d^6^ configuration of Fe^2+^, corroborating the results obtained by QTAIM (Figure 10).^66^ For the Fe–Cl interaction, the sums of all ρ(*r*) and η(*r*) values in the FeNiDES system were Σρ(*r*) = 9.402 × 10^–2^ and Ση(*r*) = 2.273 × 10^–1^; for the Ni–Cl interaction, Σρ(*r*) = 1.889 × 10^–1^ and Ση(*r*) = 3.702 × 10^–1^. For Fe–(O1, O2) interaction, the values were Σρ(*r*) = 8.530 × 10^–2^ and Ση(*r*) = 9.012 × 10^–2^ and for Ni–(O1, O2), Σρ(*r*) = 5.557 × 10^–2^ and Ση(*r*) = 5.199 × 10^–2^. Therefore, due to the greater depletion of electron density, it can be inferred that Fe–(O1, O2) interactions are stronger than Ni–(O1, O2).

Fe^2+^ and Ni^2+^ ions form coordination complexes with Cl^–^ and solvent molecules acting as ligands in their primary coordination spheres. In DES, chloride ions dominate the inner coordination sphere, while secondary interactions with solvent molecules form the outer coordination environment. The coordination number of the metal ions varies with the solvent environment, reflecting the balance between inner-sphere ligands and outer-sphere solvation. For example, Fe^2+^ shows a coordination number of four to five in DES, mainly involving Cl^–^ and ethylene glycol. At the same time, in water it forms a fully solvated octahedral complex with six ligands.^67^

#### 3.4.2 FeNiW300 and FeNiW5580

The behavior of the mixture of Fe^2+^ and Ni^2+^ ions in ethaline and water was analyzed the same way; there were also insignificant changes in the isolated ions in the same amounts of water, according to the MD results. The *g*(*r*)ρ of the FeNiWater system showed the most interactions between the Fe^2+^ ion and Cl^–^ at around 2.51–2.49 Å, followed by Ni^2+^ with the Cl^–^ ion at 2.38 Å at W300; as at W5580 the value is close to zero (Figure 9a). The other interactions were Fe-OW at around 2.19 Å and Ni-OW at around 1.96 Å (Figure 9a). A second solvation layer also appears around the peaks at 4.23 and 4.54 Å. When compared with the FeWater and NiWater systems, the CN values for FeNiW300 were equal to 1.36 Cl^–^ ions around Ni^2+^ and 2.03 Cl^–^ ions around Fe^2+^; with the oxygens of water, 4.07 water molecules around Fe^2+^ and 4.68 around Ni^2+^. For FeNiW5580, 0.14 Cl^–^ ions around Ni^2+^ and 0.84 Cl^–^ ions around Fe^2+^; with the water oxygen, 5.27 water molecules around Fe^2+^ and 5.96 around Ni^2+^, so the behavior of the ions in the mixture was similar to that of the isolated form (Figure 9b).

In contrast, in the QTAIM for the Fe–Cl interaction, the sum of all ρ(*r*) and η(*r*) values in the FeNiW300 system were Σρ(*r*) = 7.385 × 10^–2^ and Ση(*r*) = 1.847 × 10^–1^; for the Ni–Cl interaction, Σρ(*r*) = 5.860 × 10^–2^ and Ση(*r*) = 2.092 × 10^–1^. For interaction in the FeNiW5580, Fe–Cl system, the values were Σρ(*r*) = 4.374 × 10^–1^ and Ση(*r*) = 1.279 × 10^–1^ and for Ni–Cl, there are no values. Therefore, Fe–Cl has a stronger interaction than Ni–Cl due to the greater affinity of Fe^2+^ for Cl^–^ ions than Ni^2+^. On the other hand, Ni-OW is stronger than Fe-OW, indicating that Ni^2+^ has a greater affinity for forming more stable hydrated complexes with water (Figure 13). With the FeNi systems with 300 and 5580 water molecules, for Fe-OW interactions, the sums of all ρ(*r*) and η(*r*) values were FeNiW300, Σρ(*r*) = 1.259 × 10^–1^ and Ση(*r*) = 1.265 × 10^–1^; FeNiW5580, Σρ(*r*) = 5.211 × 10^–1^ and Ση(*r*) = 5.040 × 10^–1^. For Ni-OW interactions, the sums of all ρ(*r*) and η(*r*) values were FeNiW300, Σρ(*r*) = 2.079 × 10^–1^ and Ση(*r*) = 2.042 × 10^–1^; FeNiW5580, Σρ(*r*) = 4.064 × 10^–1^ and Ση(*r*) = 6.169 × 10^–1^ (Figure 10). In the mixture of metal ions, the sum of the ρ(*r*), ∇^2^ρ(*r*) and η(*r*) values for Fe–Cl in DES was lower, and the sum of the Laplacian of the electronic density for Fe–(O1, O2) also increased. At 300 and 5580 water molecules, the ρ(*r*), ∇^2^ρ(*r*), and η(*r*) values were higher for the interactions with water and the chloride ion, and there was presence of the Fe–Cl interaction for the latter amount of water, a fact that was not observed for Fe^2+^ alone. For Ni^2+^, the trend was similar except for W5580, which only showed Ni-OW interactions (Figure 10).

**Figure 11:**
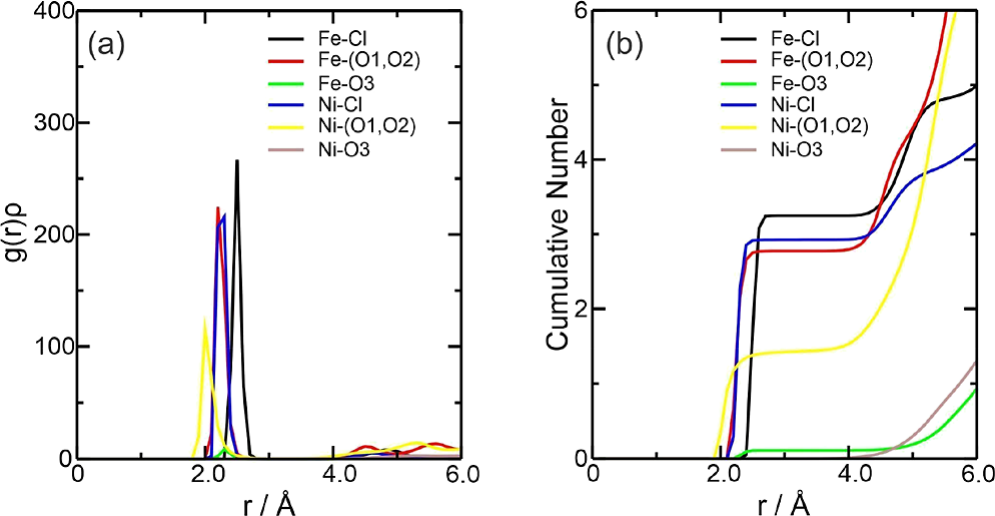
Radial distribution function multiplied by the average number density of the observed atoms, *g*(*r*)ρ, around the Fe^3+^ and Ni^2+^ ions present in the equimolar mixture (a) Fe3NiDES system and (b) CN of the counterions for this system. The color references in this figure are [(Fe3–Cl, black line), (Fe3–(O1, O2), red line), and (Fe3–O3, green line)] and [(Ni–Cl, blue line), (Ni–(O1, O2), yellow line), and (Ni–O3, brown line)].

**Figure 12:**
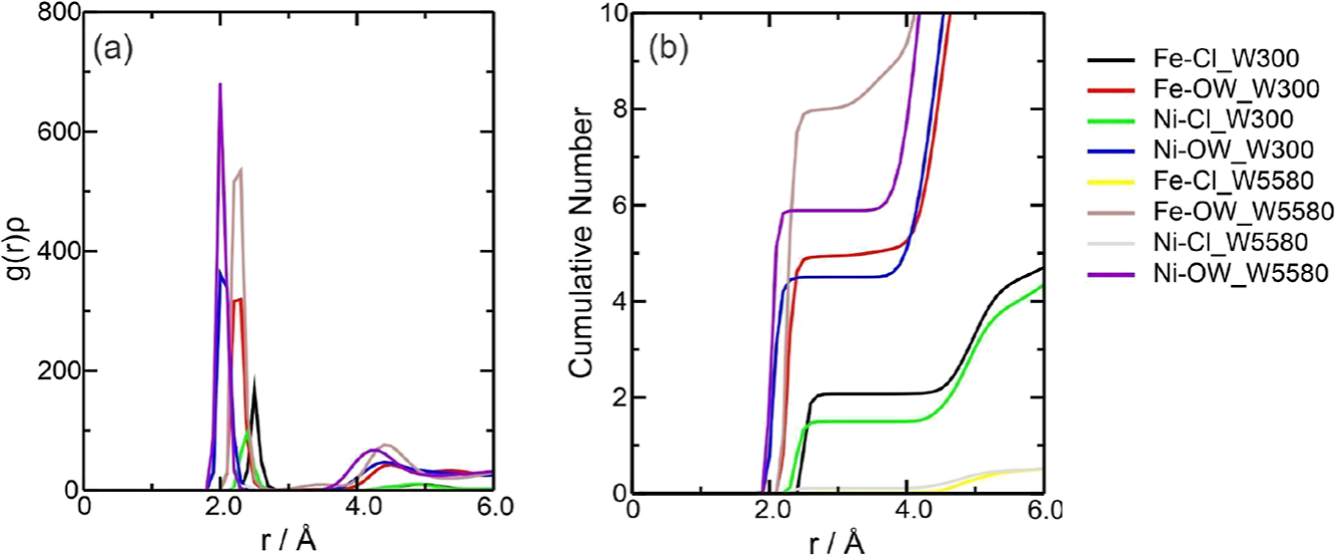
Radial distribution function multiplied by the average number density of the observed atoms, *g*(*r*)ρ, around the Fe^3+^ and Ni^2+^ ions present in the equimolar mixture (a) FeNiW*X* systems (where *X* = 300 or 5580 water molecules added) and (b) CN of the counterions for these systems. The color references in this figure were: [water 300, (Fe3–Cl, black line), (Fe3-OW, red line), (Ni–Cl, green line), (Ni-OW, blue line) and water 5580, (Fe3–Cl, yellow line), (Fe3-OW, brown line), (Ni–Cl, gray line), and (Ni-OW, violet line)].

**Figure 13:**
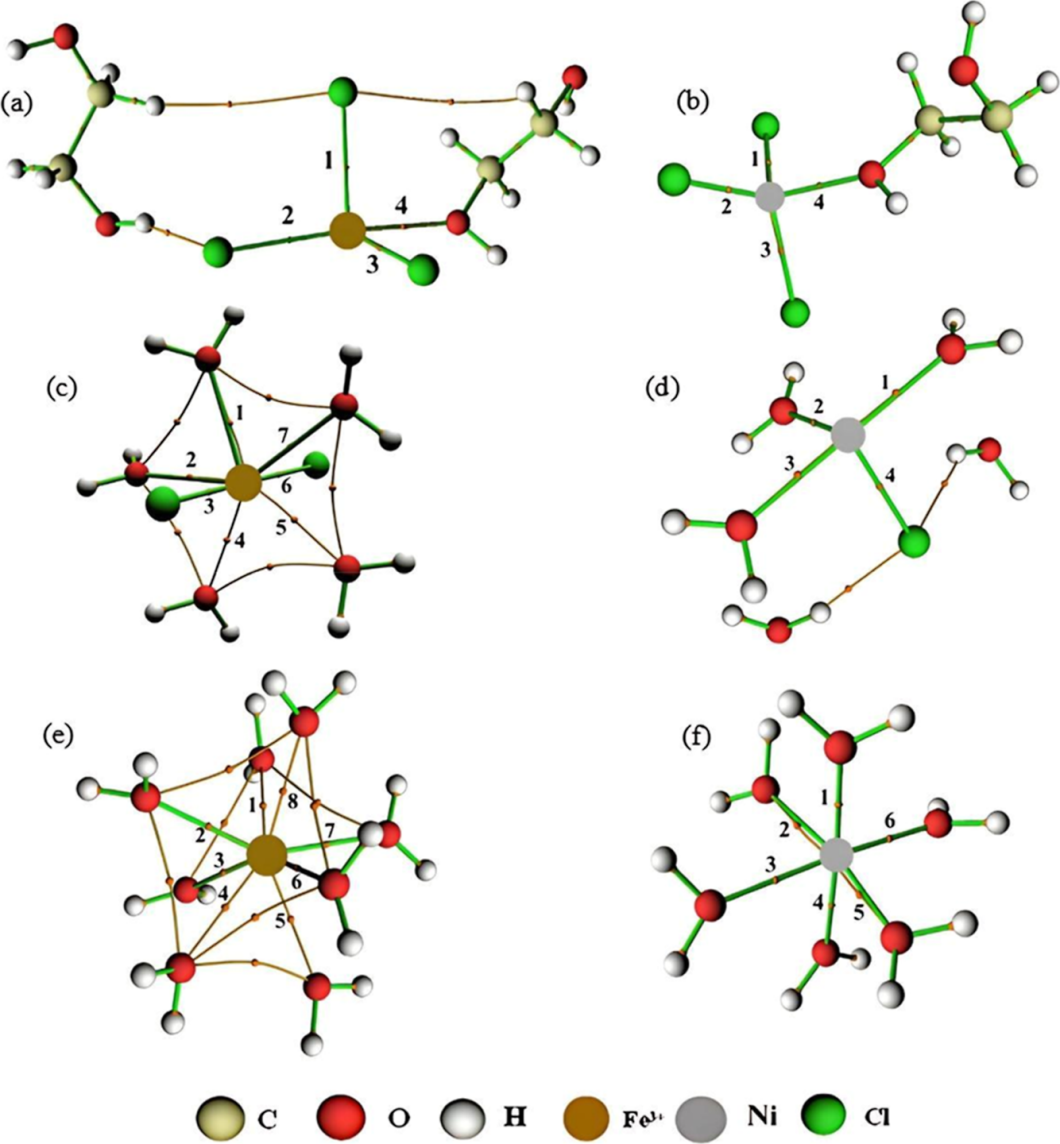
BCPs and intermolecular interactions for Fe^3+^ (a) and Ni^2+^ (b) ions with the Fe3NiDES systems; Fe^3+^ (c) and Ni^2+^ (d) ions with the Fe3NiW300 systems; Fe^3+^ (e) and Ni^2+^ (f) ions with the Fe3NiW5580 systems.

### 3.5 Fe3NiDES and Fe3NiWater (300 and 5580)

#### 3.5.1 Fe3NiDES

In the Fe3NiDES system, the *g*(*r*)ρ results showed a solvation layer and the most significant interactions were between the metal ions and the Cl^–^ anion at around 2.20 Å for Ni–Cl and 2.48 Å for Fe–Cl, followed by the Ni–(O1, O2) interactions at around 1.98 Å and Fe–(O1, O2) at around 2.20 Å (Figure 11a). According to Figure 11b, there are, on average, approximately 3.31 Cl^–^ ions and 2.82 ethylene glycol molecules complexing Fe^3+^ and 2.94 Cl^–^ ions and 1.40 ethylene glycol molecules complexing Ni^2+^. For the Fe–Cl interaction, the sums of all ρ(*r*) and η(*r*) values in the Fe3NiDES system were Σρ(*r*) = 1.261 × 10^–1^ and Ση(*r*) = 6.801 × 10^–1^; for the Ni–Cl interaction, Σρ(*r*) = 2.016 × 10^–1^ and Ση(*r*) = 3.709 × 10^–1^. For Fe–(O1, O2) interaction, the values were Σρ(*r*) = 3.806 × 10^–2^ and Ση(*r*) = 4.781 × 10^–2^ and for Ni–(O1, O2), Σρ(*r*) = 6.193 × 10^–2^ and Ση(*r*) = 5.560 × 10^–2^. Thus, by analyzing the QTAIM data, it was concluded that the iron charge increased the strength of the interactions in this mixture compared to that in the FeNiDES system. However, when dealing with the systems with the ions alone, this effect implied a decrease in the strength of these interactions for the Fe^3+^ ion and an increase for Ni^2+^ (Figure 13).

#### 3.5.2 Fe3NiWater (300 and 5580)

In the presence of water, the *g*(*r*)ρ results showed two solvation layers approximately between 1.96 and 2.47 and 4.19–4.49 Å. Interactions between metal ions and the Cl^–^ anion were observed for only 300 water molecules. In contrast, the Fe3-OW and Ni-OW interactions were strong for Fe^3+^ and Ni^2+^ (Figure 12a). Looking at the CN (Figure 12b), the Fe3NiW300 system has approximately 2.15 Cl^–^ ions, 4.96 water molecules around Fe^3+^, and 1.52 Cl^–^ ions and 4.51 water molecules around Ni^2+^. In Fe3NiW5580, no interactions with Cl^–^ ions are detected for the two metal species, but there are around 7.97 water molecules around Fe^3+^ and 5.90 water molecules around Ni^2+^. For the Fe–Cl interaction, the sums of all ρ(*r*) and η(*r*) values in the Fe3NiW300 system were Fe3NiW300, Σρ(*r*) = 8.822 × 10^–2^ and Ση(*r*) = 2.318 × 10^–1^; Fe3NiW5580, showed no values for Fe–Cl and Ni–Cl. The Fe3NiW300 system showed Σρ(*r*) = 1.650 × 10^–1^ and Ση(*r*) = 2.291 × 10^–1^ for Fe3-OW and Σρ(*r*) = 1.655 × 10^–1^ and Ση(*r*) = 1.427 × 10^–1^ for Ni-OW. The Fe3NiW5580 system showed Σρ(*r*) = 2.911 × 10^–1^ and Ση(*r*) = 4.281 × 10^–1^ for Fe3-OW and Σρ(*r*) = 4.127 × 10^–1^ and Ση(*r*) = 3.529 × 10^–1^ for Ni-OW. Therefore, the tendency observed in water was the formation of hydrated complexes with the metal ions, with Fe^3+^ being more favored due to its higher charge density. However, upon comparison of these systems with the isolated ions, the effect observed was similar to that of mixtures of systems with Fe^2+^ and Ni^2+^ (Figure 13).

It is important to highlight in the literature the work of Bryantsev et al., which deals with studying the hydration sphere of the Cu^2+^ ion by using DFT calculations combined with the COSMO continuous solvent model. The authors analyzed the influence of the coordination number (4 to 6) on the structure and stability of the [Cu(H_2_O)_*n*_]^2+^ complexes; finding that, in the gas phase, more open structures are favored, while, in aqueous solution, compact configurations, such as the square pyramidal geometry with five coordinations, become more stable due to electrostatic interactions.^68^ The first layer comprises water molecules that directly coordinate with the metal ion. In contrast, the second layer consists of water molecules that interact more weakly with the ion yet still play a significant role in modulating its properties. These theoretical results align with experimental data from extended X-ray absorption fine structure and X-ray absorption near edge structure, validating the predictions about hydration dynamics and providing relevant insights into the interactions of Cu^2+^ in aqueous media.^68,69^

### 3.6 NCI/RDG

**Figure 14:**
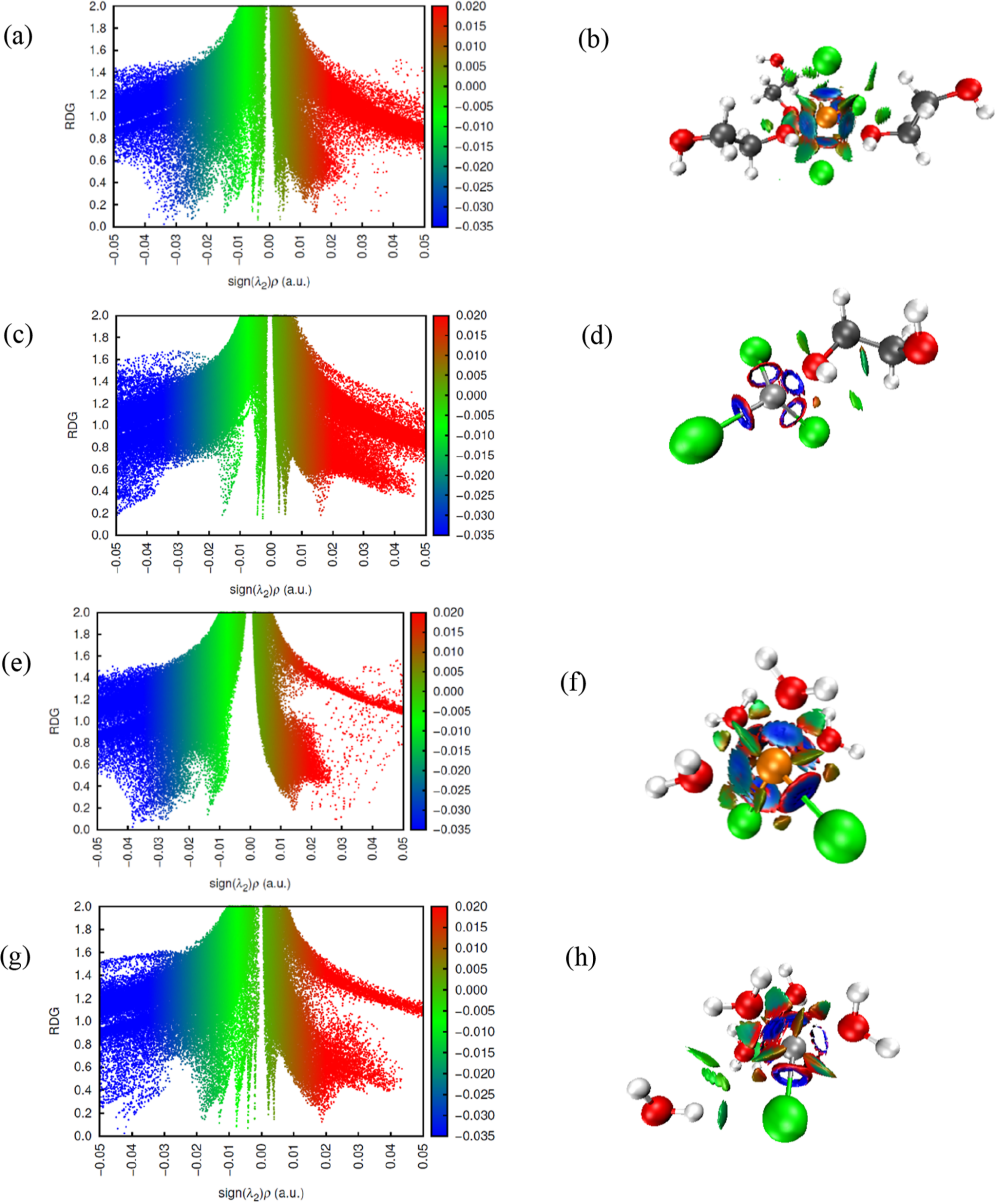
RDG vs sign(λ_2_)ρ from the results of the NCI and QTAIM analyses for Fe^2+^ ion in FeNiDES system (a) and intermolecular interactions for the species (b); Ni^2+^ ion in FeNiDES system (c) and intermolecular interactions for the species (d); Fe^2+^ ion in FeNiW300 system (e) and intermolecular interactions for the species (f); Ni^2+^ ion in FeNiW300 system (g) and intermolecular interactions for the species (h).

**Figure 15:**
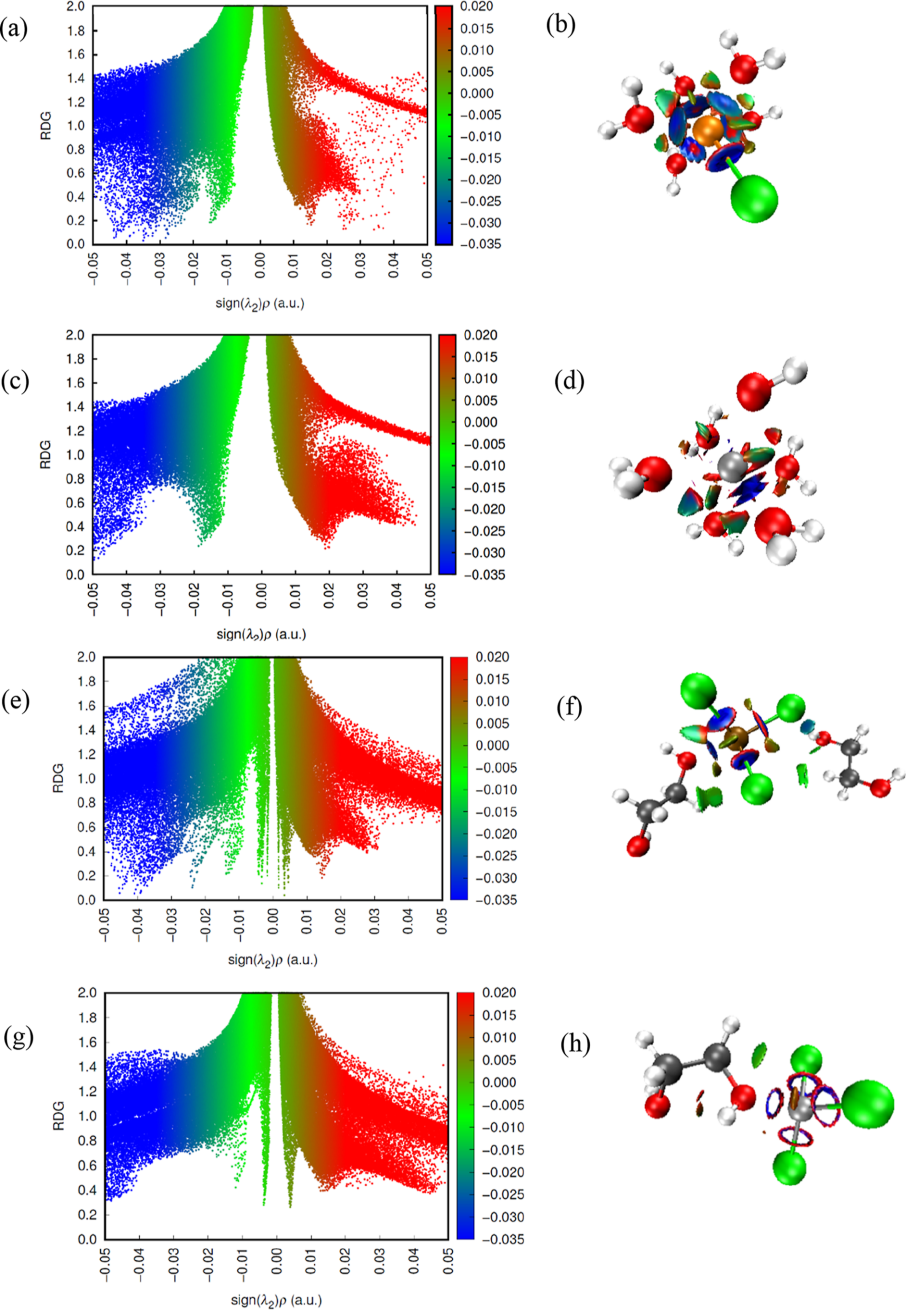
RDG vs sign(λ_2_)ρ from the results of the NCI and QTAIM analyses for Fe^2+^ ion in FeNiW5580 system (a) and intermolecular interactions for the species (b); Ni^2+^ ion in FeNiW5580 system (c) and intermolecular interactions for the species (d); Fe^3+^ ion in Fe3NiDES system (e) and intermolecular interactions for the species (f); Ni^2+^ ion in Fe3NiDES system (g) and intermolecular interactions for the species (h).

**Figure 16:**
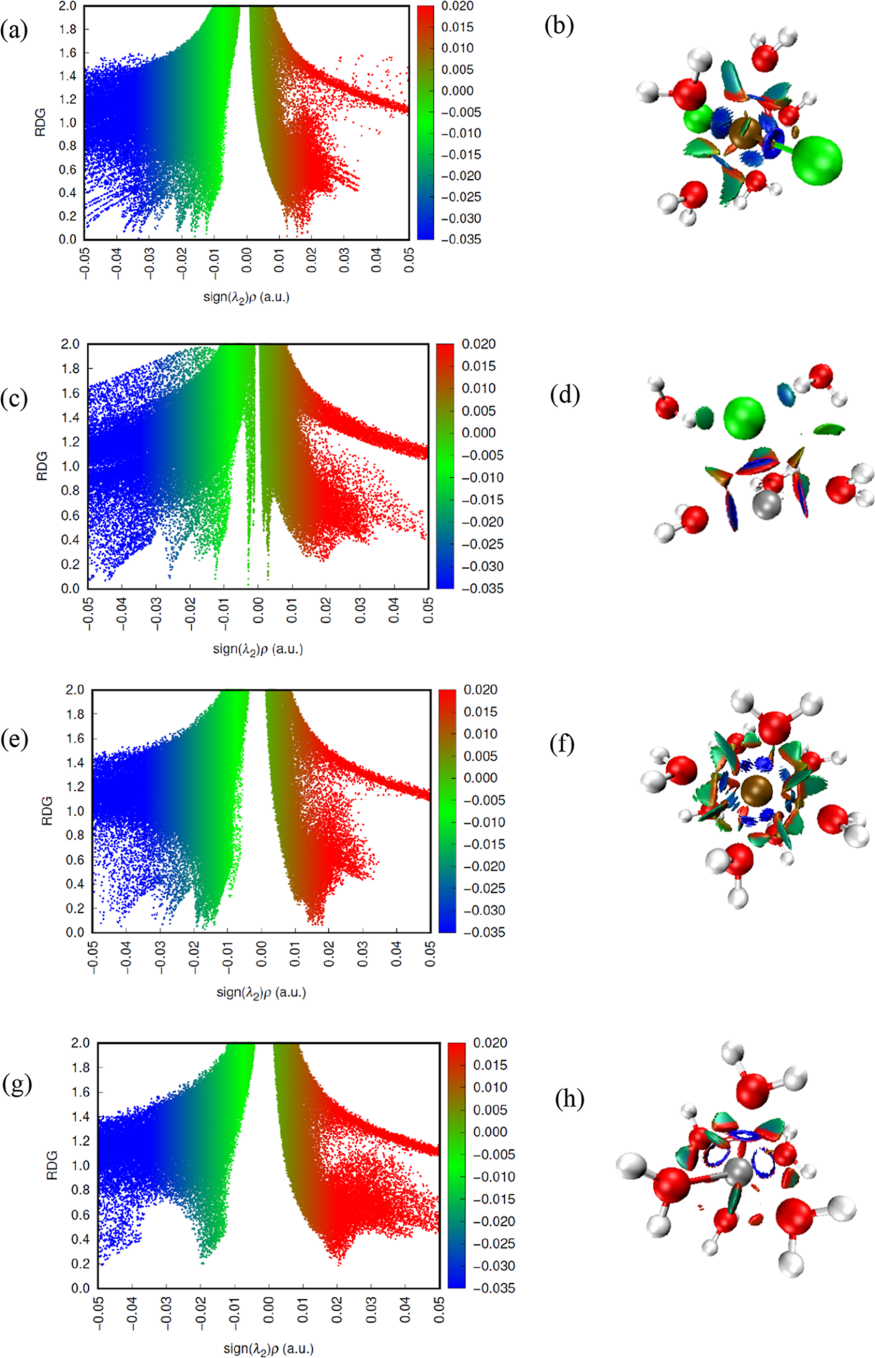
RDG vs sign(λ_2_)ρ from the results of the NCI and QTAIM analyses for Fe^3+^ ion in Fe3NiW300 system (a) and intermolecular interactions for the species (b); Ni^2+^ ion in Fe3NiW300 system (c) and intermolecular interactions for the species (d); Fe^3+^ ion in Fe3NiW5580 system (e) and intermolecular interactions for the species (f); Ni^2+^ ion in Fe3NiW5580 system (g) and intermolecular interactions for the species (h).

The NCI maps showed the behavior of the metal ions in isolation and in equimolar mixtures in ethaline and water (Figures 14 to 16 and Supporting Information). The results showed that the Fe^2+^ and Fe^3+^ ions behaved differently in isolation so that the 3+ ion showed less dispersion in all regions in both water and DES, with more defined peaks in the low RDG areas and negative values of (sign λ_2_)ρ, which corroborates the fact that it is more solvated when in the presence of water. The colors representing the elements or ions were Fe^2+^ (orange), Ni^2+^ (silver), Fe^3+^ (ochre), chlorine (Cl, green), oxygen (O, red), carbon (C, gray), and hydrogen (H, white).

#### 3.6.1 Fe3DES and Fe3Water (300 and 5580) Systems

For the system with Fe^3+^ in DES, the distribution is similar to that of iron, showing a distribution with around 4 well-defined peaks in the green region, 2 in the red area, and 2 in the blue region. Due to its greater charge and reactivity, the Fe^3+^ ion showed more intense interactions in the blue region than the Fe^2+^ ion. As for the Fe3Water systems, in the presence of 5580 water molecules, there are around 3 peaks in the −0.02 sign < (λ_2_)ρ < 0.01 region, indicating the presence of weaker interactions compared to 300-molecule systems due to the greater dilution. There was an increase in dispersion in the region 0.01 sign < (λ_2_)ρ < 0.03, which may be related to repulsions caused by hydrogen bonds.

#### 3.6.2 NiDES and NiWater (300 and 5580) Systems

An evolution in NCI was observed as the system with Ni^2+^ changed from DES to W300 and W5580. This reflects the changes in the solvation structure of Ni^2+^ in different environments, from a eutectic solvent to more dilute aqueous system (W300 and W5580). The graphs corroborate this interpretation, showing different arrangements of molecules around the Ni^2+^ ion, transitioning from a more compact environment in DES to a more dispersed environment in the aqueous systems, especially in W5580. In DES, there are 3 well-defined peaks in the green region and 1 in the blue and red regions. In NiWater systems, in the presence of 300 water molecules, there were around 3 peaks in the −0.01 sign < (λ_2_)ρ < 0.01 region, characterizing weak interactions, while in 5580 water molecules, there is 1 peak in each region of the graph, which implies the effects of dilution, ion–dipole interactions, and hydrogen bonds, mapped in attractive and repulsive regions.

#### 3.6.3 FeNiDES and FeNiWater (300 and 5580) Systems

In the case of mixed systems such as Fe^2+^ and Ni^2+^, there may be competition between Fe and Ni for the solvent molecules, altering the NCI patterns compared to those of single metal ion systems. It can be seen especially for Ni^2+^, whose behavior differed little in the mixture with the disappearance of peaks in the weaker regions and 1 well-defined peak in the region at −0.02 sign < (λ_2_)ρ < 0.01 (blue) and 1 peak at 0.01 sign < (λ_2_)ρ < 0.02 (red), which is justified by the values of the properties obtained by QTAIM especially in the Ni-(O1, O2) interaction that only appears in the mixture of ions.

#### 3.6.4 Fe3NiDES and Fe3NiWater (300 and 5580) Systems

In the mixture of Fe^3+^ and Ni^2+^ ions in DES, the Fe3–Cl interaction was more present, with around 4 peaks in the blue region. The weak interactions also showed 4 peaks at −0.01 sign < (λ_2_)ρ < 0.01. The graphs show similar patterns to the systems with only one type of ion, indicating that the intermolecular interactions are not significantly affected by the presence of both ions. The blue, green, and red regions indicate the same interactions observed in the systems with a single ion. In the Fe3NiW300 system, the attractive interactions (blue) are more intense than in Fe3NiW5580, indicating a more compact solvation structure and diffuse and less intense interactions in the more dilute system. An increase in repulsive interactions (red) and greater dispersion of van der Waals interactions (green) with Fe^3+^, which has more water molecules around it, were also observed. NCI plots corroborate this interpretation, evidenced by the CN, showing a more widely spaced distribution of water molecules around the ions in the W5580 system compared to the W300.

## 4 Conclusions

As expected, systems with a higher water content have strong hydrogen bonds, as seen in the blue region of the NCI maps. Considering that the solvents used in this work were ethaline and water, the intermolecular forces presented here were studied for comparison. In the systems with 5580 water molecules, the metal ions were surrounded by water molecules clustered around them, forming a hydration layer and characterizing an ion–dipole interaction. For the species isolated in water, the behavior showed more water molecules around the Fe^3+^ ions in both quantities of water (W300 and W5580), followed by Fe^2+^ and Ni^2+^ for W300. For the system with W5580, the patterns of Fe^2+^ and Ni^2+^ ions was similar.

The ability of an ion in solution to distort the electron cloud of its neighboring molecules is related to its polarizability, mainly depending on the size of the ion and its effective nuclear charge. The charges of the ions were considered to compare the polarizability of the metal ions in both water and ethaline and rank them in an ascending order. Fe^2+^ and Ni^2+^ have a +2 charge, and Fe^3+^ has a +3 charge, while the size of the ionic radii corresponded to Fe^2+^: 78 pm, Fe^3+^: 65 pm, and Ni^2+^: 69 pm. Therefore, the increasing order of polarizability is Fe^3+^ < Ni^2+^ < Fe^2+^, with Fe^3+^ being the least polarizable due to its high charge and smaller ionic radius, followed by Ni^2+^ and Fe^2+^ as the most polarizable of the three due to its larger size among the +2 charged ions. In ethaline, some properties, such as its lower polarity and dielectric constant than water and its higher viscosity, can result in less shielding of the species’ charges and affect ion mobility. Despite this, the order of polarizability will probably remain similar to that observed in water but with possible changes in the relative magnitude.

The electron density [ρ] and ∇^2^ρ(*r*) values in the BCPs were higher for the bonds between the metal ions and water. The results revealed that Fe is more reactive, oxidizing faster than Ni. Both elements have similar ionization energies, although Fe has a slightly higher value, and Ni is more electronegative. MD simulations and QTAIM calculations provided an understanding of the interactions between metal ions and their ligands. The results indicated that, in aqueous solution systems, Fe^3+^ and Fe^2+^ ions form distinct hydration layers, with Fe^3+^ showing more intense interactions due to its greater charge. In DES, the interactions between the ions and the solvent were characterized by stronger bonds in the case of Ni^2+^ with Cl^–^ compared to Fe^2+^. Overall, this study can serve as a basis for research and practical applications in corrosion, electrodeposition, and catalysis.

## Supporting Information Available

Detailed tables with topological analyses obtained using the QTAIM method, describing BCPs and molecular interactions involving Fe^2+^, Fe^3+^, and Ni^2+^ ions in systems composed of ethaline and water; values of electronic density, Laplacian of the electronic density, and the ELF index are presented for different interactions, such as Fe–Cl, Fe–(O1,O2), Fe-OW, Ni–Cl, Ni–(O1,O2), and Ni-OW, for different solvent conditions; and NCI (non-covalent interactions) maps and RDG graphs, which help to visualize the behavior of the metal ions in isolation and equimolar mixtures in the systems evaluated (PDF)
