# Supplementary material for: Theoretical Study of Fe3+ and Ni2+ Ion Interactions in Ethaline as the Deep Eutectic Solvent and Water Solutions Using Molecular Dynamics, Quantum Theory of Atoms in Molecules, and Non-Covalent Interactions
Source: ACS Omega. 2025 Apr 16;10(16):16015–30. doi: 10.1021/acsomega.4c08992 (PMC12044496; doi:10.1021/acsomega.4c08992)
Supplement: Supplementary file 1 — ao4c08992_si_001.pdf [file ao4c08992_si_001.pdf]

ACS Omega

Supporting Information

Theoretical Study of Fe^{3+} and Ni^{2+} Ions Interactions in Ethaline as the Deep Eutectic Solvent and Water Solutions Using Molecular Dynamics, Quantum Theory of Atoms in Molecules and Non-Covalent Interactions

Laudenor Amorim[a], Renato Veríssimo de Oliveira[a], Lucas Lima Bezerra[a], Pierre Basílio Almeida Fechine[a,b], Adriana Nunes Correia[a], Pedro de Lima-Neto[a], Norberto de Kássio Vieira Monteiro[a*].

[a]Department of Analytical Chemistry and Physical Chemistry, Science Center, Federal University of Ceará, Pici Campus, Block 940, 60440-900, Fortaleza, CE, Brazil.

[b]Department of Metallurgical and Materials Engineering, Technology Center, Federal University of Ceará, Pici Campus, Block 729, 60440-554, Fortaleza, CE, Brazil.

*Corresponding author.

E-mail address: norbertokv@ufc.br (Norberto K. V. Monteiro).

The tables in this supporting information represent the Bond Critical Points (BCPs) obtained by QTAIM, highlighting the molecular interactions between the metal ions in ethaline and water.

Table S1: Topological analysis obtained by QTAIM between the Fe^{3+} ions with FeDES and FeWX systems (where X= 300 or 5580 water molecules added). Electron density, *ρ(r),* laplacian of electron density, $\nabla^2\rho(r)$ and ELF value, *η(r)* at a bond critical point (BCP) of selected Fe-Cl, Fe-(O1, O2), and Fe-OW interactions.

Interaction	BCP	*ρ(r)*	$\nabla^2\rho(r)$	*η(r)*	System
Fe-Cl	P_1	3.592×10^{-2}	1.368×10^{-1}	1.218×10^{-1}	
	P_2	3.957×10^{-2}	1.542×10^{-1}	1.214×10^{-1}	
	P_4	3.662×10^{-2}	1.417×10^{-1}	1.152×10^{-1}	
	P_5	3.254×10^{-2}	1.122×10^{-1}	1.159×10^{-1}	
Σ		1.4		4.743×10^{-1}	Fe3DES
Fe-(O1,O2)	P_3	3.277×10^{-2}	2.384×10^{-1}	2.989×10^{-2}	
	P_6	2.603×10^{-2}	1.886×10^{-1}	2.296×10^{-2}	
Σ		5.880×10^{-2}		5.285×10^{-2}	
Fe-Cl	P_3	4.046×10^{-2}	1.629×10^{-1}	1.269×10^{-2}	
	P_7	4.350×10^{-2}	1.753×10^{-1}	1.393×10^{-1}	
Σ		8.396×10^{-2}		1.520×10^{-1}	
Fe-OW	P_1	3.669×10^{-2}	2.399×10^{-1}	5.338×10^{-2}	Fe3W300
	P_2	3.980×10^{-2}	2.819×10^{-1}	4.908×10^{-1}	
	P_4	3.370×10^{-2}	2.159×10^{-1}	5.071×10^{-2}	
	P_5	3.826×10^{-2}	2.624×10^{-1}	4.990×10^{-2}	
	P_6	3.065×10^{-2}	1.986×10^{-1}	4.433×10^{-2}	
Σ		1.791×10^{-1}		6.891×10^{-1}	
Fe-OW	P_1	4.086×10^{-2}	2.274×10^{-1}	6.974×10^{-2}	Fe3W5580
	P_2	3.889×10^{-2}	2.178×10^{-1}	6.960×10^{-2}	
	P_3	3.825×10^{-2}	2.054×10^{-1}	6.959×10^{-2}	
	P_4	4.186×10^{-2}	2.307×10^{-1}	7.366×10^{-2}	
	P_5	4.355×10^{-2}	2.323×10^{-1}	7.919×10^{-1}	

	P_1	4.086×10^{-2}	2.274×10^{-1}	6.974×10^{-2}	
	P_6	3.384×10^{-2}	1.740×10^{-1}	6.871×10^{-2}	
	P_7	3.294×10^{-2}	1.870×10^{-1}	5.742×10^{-2}	
Σ		2.702×10^{-1}		1.201	

Table S2: Topological analysis obtained by QTAIM between the Ni^{2+} ions with NiDES and NiWX systems (where X= 300 or 5580 water molecules added). Electron density, *ρ(r),* laplacian of electron density, $\nabla^2\rho(r)$ and ELF value, *η(r)* at a bond critical point (BCP) of selected Ni-Cl, Ni-(O1, O2), and Ni-OW interactions.

Interaction	BCP	$\rho(r)$	$\nabla^2\rho(r)$	$\eta(r)$	System
Ni-Cl	P_1	4.664×10^{-2}	2.078×10^{-1}	1.102×10^{-1}	NiDES
	P_2	7.408×10^{-2}	2.946×10^{-1}	1.720×10^{-1}	
	P_3	6.673×10^{-2}	3.569×10^{-1}	1.085×10^{-1}	
Σ		1.875×10^{-1}		3.907×10^{-1}	
Ni-Cl	P_3	5.512×10^{-2}	1.451×10^{-1}	2.681×10^{-1}	NiW300
Σ		5.512×10^{-2}		2.681×10^{-1}	
Ni-OW	P_1	5.633×10^{-2}	4.464×10^{-1}	5.499×10^{-2}	
	P_2	6.518×10^{-2}	5.081×10^{-1}	6.284×10^{-2}	
Σ		1.215×10^{-1}		1.178×10^{-1}	
Ni-OW	P_1	5.808×10^{-2}	4.626×10^{-1}	5.601×10^{-2}	NiW5580
	P_2	6.939×10^{-2}	5.381×10^{-1}	6.261×10^{-2}	
	P_3	6.288×10^{-2}	4.764×10^{-1}	5.919×10^{-2}	
	P_4	6.028×10^{-2}	4.918×10^{-1}	5.554×10^{-2}	
	P_5	5.120×10^{-2}	3.824×10^{-1}	5.370×10^{-2}	
	P_6	5.322×10^{-2}	4.026×10^{-1}	5.082×10^{-2}	
Σ		3.551×10^{-1}		3.379×10^{-1}	

Table S3: Topological analysis obtained by QTAIM between the Fe^{2+} and Ni^{2+} ions with FeNiDES and FeNiWX systems (where X= 300 or 5580 water molecules added). Electron density, *ρ(r),* laplacian of electron density, $\nabla^2\rho(r)$ and ELF value, *η(r)* at a bond critical point (BCP) of selected Fe-Cl, Fe-(O1, O2), Fe-OW, Ni-Cl, Ni-(O1, O2) and Ni-OW interactions.

Interaction	BCP	*ρ(r)*	$\nabla^2\rho(r)$	*η(r)*	System
Fe-Cl	P_1	3.392×10^{-2}	1.472×10^{-1}	7.404×10^{-2}	FeNiDES
	P_2	2.904×10^{-2}	1.200×10^{-1}	7.290×10^{-2}	
	P_5	3.106×10^{-2}	1.266×10^{-1}	8.033×10^{-2}	
Σ		9.402×10^{-2}		2.273×10^{-1}	
Fe-(O1,O2)	P_3	3.885×10^{-2}	2.803×10^{-1}	3.799×10^{-2}	
	P_4	2.621×10^{-2}	1.817×10^{-1}	2.583×10^{-2}	
	P_6	2.524×10^{-2}	1.699×10^{-1}	2.630×10^{-2}	
Σ		8.530×10^{-2}		9.012×10^{-2}	
Ni-Cl	P_1	6.757×10^{-2}	3.203×10^{-1}	1.296×10^{-1}	
	P_2	5.429×10^{-2}	2.791×10^{-1}	9.812×10^{-2}	
	P_3	6.701×10^{-2}	2.951×10^{-1}	1.425×10^{-1}	
Σ		1.889×10^{-1}		3.702×10^{-1}	
Ni-(O1,O2)	P_4	5.557×10^{-2}	4.540×10^{-1}	5.199×10^{-2}	
Σ		5.557×10^{-2}		5.199×10^{-2}	
	P_1	3.856×10^{-2}	1.624×10^{-1}	8.611×10^{-2}	FeNiW300
Fe-Cl	P_2	3.529×10^{-2}	1.344×10^{-1}	9.855×10^{-2}	
Σ		7.385×10^{-2}		1.847×10^{-1}	
Fe-OW	P_3	3.283×10^{-2}	2.352×10^{-1}	3.137×10^{-2}	
	P_4	2.771×10^{-2}	1.835×10^{-1}	3.109×10^{-2}	
	P_5	2.960×10^{-2}	2.168×10^{-1}	2.639×10^{-2}	
	P_6	3.576×10^{-2}	2.498×10^{-1}	3.766×10^{-2}	
Σ	4	1.259×10^{-1}		1.265×10^{-1}	
Ni-Cl	P_5	5.860×10^{-2}	1.888×10^{-1}	2.092×10^{-1}	
Σ	1	5.860×10^{-2}		2.092×10^{-1}	
Ni-OW	P_1	5.775×10^{-2}	4.434×10^{-1}	5.484×10^{-2}	
	P_2	4.249×10^{-2}	3.116×10^{-1}	4.337×10^{-2}	
	P_3	6.291×10^{-2}	4.921×10^{-1}	5.702×10^{-2}	

	P_4	4.479×10^{-2}	3.361×10^{-1}	4.894×10^{-2}	
Σ		2.079×10^{-1}		2.042×10^{-1}	
Fe-Cl	P_1	4.374×10^{-1}	1.551×10^{-1}	1.279×10^{-1}	FeNiW5580
Σ		4.374×10^{-1}	1.551×10^{-1}	1.279×10^{-1}	
Fe-OW	P_2	2.842×10^{-2}	1.944×10^{-1}	2.902×10^{-2}	
	P_3	3.360×10^{-2}	2.263×10^{-1}	3.719×10^{-1}	
	P_4	3.504×10^{-2}	2.734×10^{-1}	2.800×10^{-2}	
	P_5	3.914×10^{-1}	2.837×10^{-1}	3.789×10^{-2}	
	P_6	3.260×10^{-2}	2.172×10^{-1}	3.723×10^{-2}	
Σ		5.211×10^{-1}		5.040×10^{-1}	
Ni-OW	P_1	7.777×10^{-2}	4.979×10^{-1}	9.747×10^{-2}	
	P_2	7.140×10^{-2}	5.043×10^{-1}	8.342×10^{-2}	
	P_3	6.310×10^{-2}	3.884×10^{-1}	9.434×10^{-2}	
	P_4	6.946×10^{-2}	4.154×10^{-1}	1.021×10^{-1}	
	P_5	7.890×10^{-2}	4.224×10^{-1}	1.575×10^{-1}	
	P_6	4.575×10^{-2}	2.574×10^{-1}	8.202×10^{-2}	
Σ		4.064×10^{-1}		6.169×10^{-1}	

Table S4: Topological analysis obtained by QTAIM between the Fe^{3+} and Ni^{2+} ions with Fe3NiDES and Fe3NiWX systems (where X= 300 or 5580 water molecules added). Electron density, $\rho(r)$, laplacian of electron density, $\nabla^2\rho(r)$ and ELF value, $\eta(r)$ at a bond critical point (BCP) of selected Fe-Cl, Fe-(O1, O2), Fe-OW, Ni-Cl, Ni-(O1, O2) and Ni-OW interactions.

Interaction	BCP	$\rho(r)$	$\nabla^2\rho(r)$	$\eta(r)$	System
Fe-Cl	P_1	4.558×10^{-2}	1.094×10^{-1}	2.792×10^{-1}	Fe3NiDES
	P_2	4.101×10^{-2}	1.259×10^{-1}	1.770×10^{-1}	
	P_3	3.952×10^{-2}	1.067×10^{-1}	2.239×10^{-1}	
Σ		1.261×10^{-1}		6.801×10^{-1}	
Fe-(O1,O2)	P_4	3.806×10^{-2}	2.426×10^{-1}	4.781×10^{-2}	
Σ		3.806×10^{-2}		4.781×10^{-2}	
Ni-Cl	P_1	6.383×10^{-2}	3.024×10^{-1}	1.248×10^{-1}	
	P_2	6.856×10^{-2}	3.810×10^{-1}	1.037×10^{-1}	
	P_3	6.921×10^{-2}	3.073×10^{-1}	1.424×10^{-1}	
Σ		2.016×10^{-1}		3.709×10^{-1}	
Ni-(O1,O2)	P_4	6.193×10^{-2}	5.108×10^{-1}	5.560×10^{-2}	
Σ		6.193×10^{-2}		5.560×10^{-2}	
Fe-Cl	P_3	5.044×10^{-2}	1.444×10^{-1}	2.269×10^{-2}	Fe3NiW300
	P_6	3.778×10^{-2}	1.035×10^{-1}	2.091×10^{-1}	
Σ		8.822×10^{-2}		2.318×10^{-1}	
Fe-OW	P_1	3.532×10^{-2}	2.199×10^{-1}	4.666×10^{-2}	
	P_2	3.524×10^{-2}	2.241×10^{-1}	4.919×10^{-2}	
	P_4	3.328×10^{-2}	2.058×10^{-1}	4.847×10^{-2}	
	P_5	2.830×10^{-2}	1.716×10^{-1}	3.997×10^{-2}	
	P_7	3.290×10^{-2}	1.968×10^{-1}	4.477×10^{-2}	
Σ		1.650×10^{-1}		2.291×10^{-1}	
Ni-Cl	P_4	4.509×10^{-2}	1.467×10^{-1}	1.806×10^{-1}	
Σ		4.509×10^{-2}		1.806×10^{-1}	
Ni-OW	P_1	4.437×10^{-2}	3.381×10^{-1}	4.048×10^{-2}	
	P_2	7.131×10^{-2}	6.043×10^{-1}	5.986×10^{-2}	

	P_3	4.978×10^{-2}	3.966×10^{-1}	4.237×10^{-2}	
Σ		1.655×10^{-1}		1.427×10^{-1}	
	P_1	2.730×10^{-2}	1.628×10^{-1}	4.518×10^{-2}	
Fe-OW	P_2	4.154×10^{-2}	2.829×10^{-1}	5.637×10^{-2}	
	P_3	4.785×10^{-2}	3.139×10^{-1}	6.574×10^{-2}	
	P_4	2.716×10^{-2}	1.635×10^{-1}	4.501×10^{-2}	
	P_5	2.951×10^{-2}	1.788×10^{-1}	4.776×10^{-2}	
	P_6	4.285×10^{-2}	2.779×10^{-1}	6.256×10^{-2}	
	P_7	4.265×10^{-2}	2.903×10^{-1}	5.836×10^{-2}	
	P_8	3.219×10^{-2}	2.110×10^{-1}	4.708×10^{-2}	
Σ		2.911×10^{-1}		4.281×10^{-1}	Fe3NiW5580
	P_1	7.692×10^{-2}	6.279×10^{-1}	6.525×10^{-2}	
	P_2	6.002×10^{-2}	4.677×10^{-1}	5.315×10^{-2}	
	P_3	5.287×10^{-2}	4.120×10^{-1}	5.339×10^{-2}	
	P_4	6.014×10^{-2}	4.721×10^{-1}	5.715×10^{-2}	
Ni-OW	P_5	8.044×10^{-2}	6.919×10^{-1}	5.879×10^{-2}	
	P_6	8.228×10^{-2}	7.044×10^{-1}	6.515×10^{-2}	
Σ		4.127×10^{-1}		3.529×10^{-1}	

Next, the NCI maps with the behavior of the metal ions in isolation and equimolar mixtures in ethaline and water (Fig S1 to S3). The colors representing the elements or ions were Fe^{2+} (orange), Ni^{2+} (silver), Fe^{3+} (ochre), chlorine (Cl, green), oxygen (O, red), carbon (C, gray) and hydrogen (H, white).

(a)

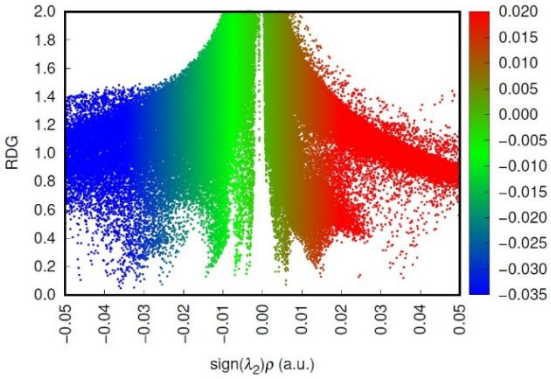

(b)

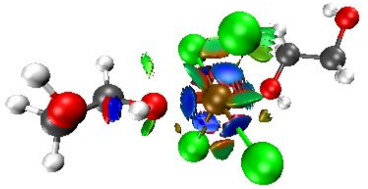

(c)

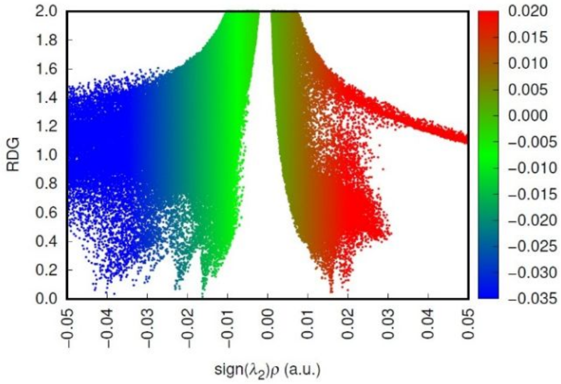

(d)

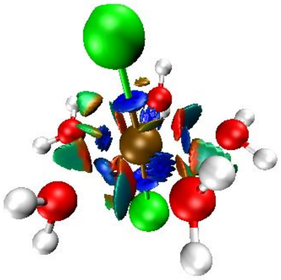

(e)

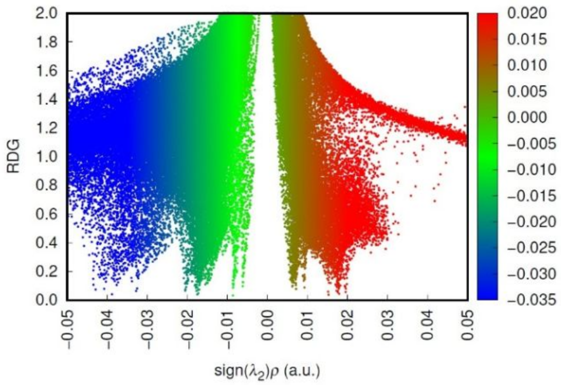

(f)

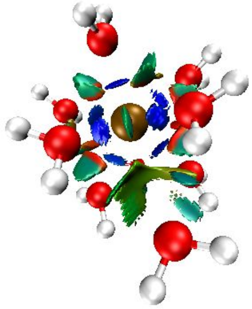

Figure S1: Reduced density gradient (RDG) vs. sign(λ_2)ρ from the results of the NCI and QTAIM analyses for Fe^{3+} ion in Fe3DES system (a) and intermolecular interactions for the species (b); Fe^{3+} ion in Fe3W300 system (c) and intermolecular interactions for the species (d); Fe^{3+} ion in Fe3W5580 system (e) and intermolecular interactions for the species (f).

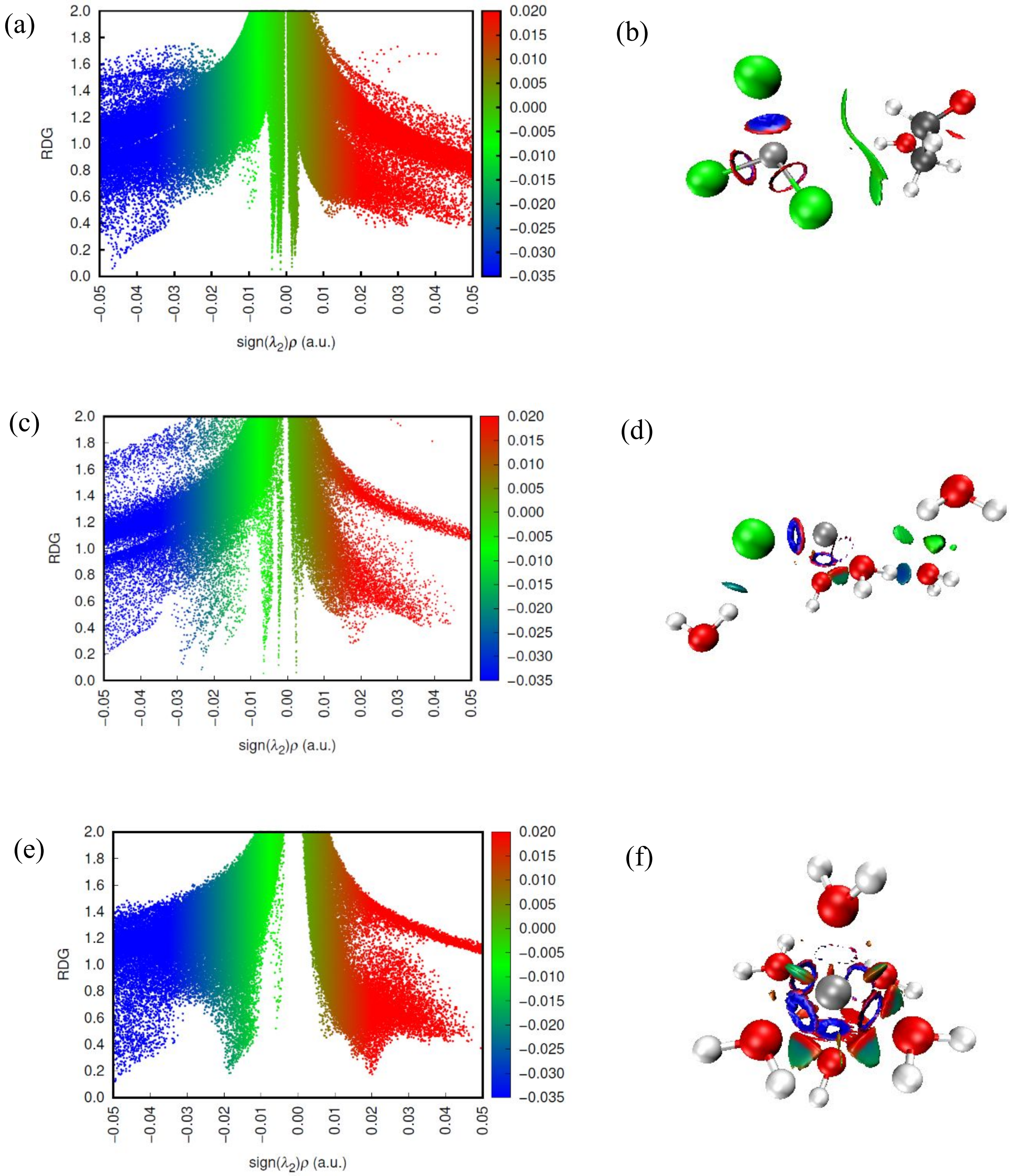

Figure S2: Reduced density gradient (RDG) vs. sign(λ_2)ρ from the results of the NCI and QTAIM analyses for Ni^{2+} ion in NiDES system (a) and intermolecular interactions for the species (b); Ni^{2+} ion in NiW300 system (c) and intermolecular interactions for the species (d); Ni^{2+} ion in NiW5580 system (e) and intermolecular interactions for the species (f).

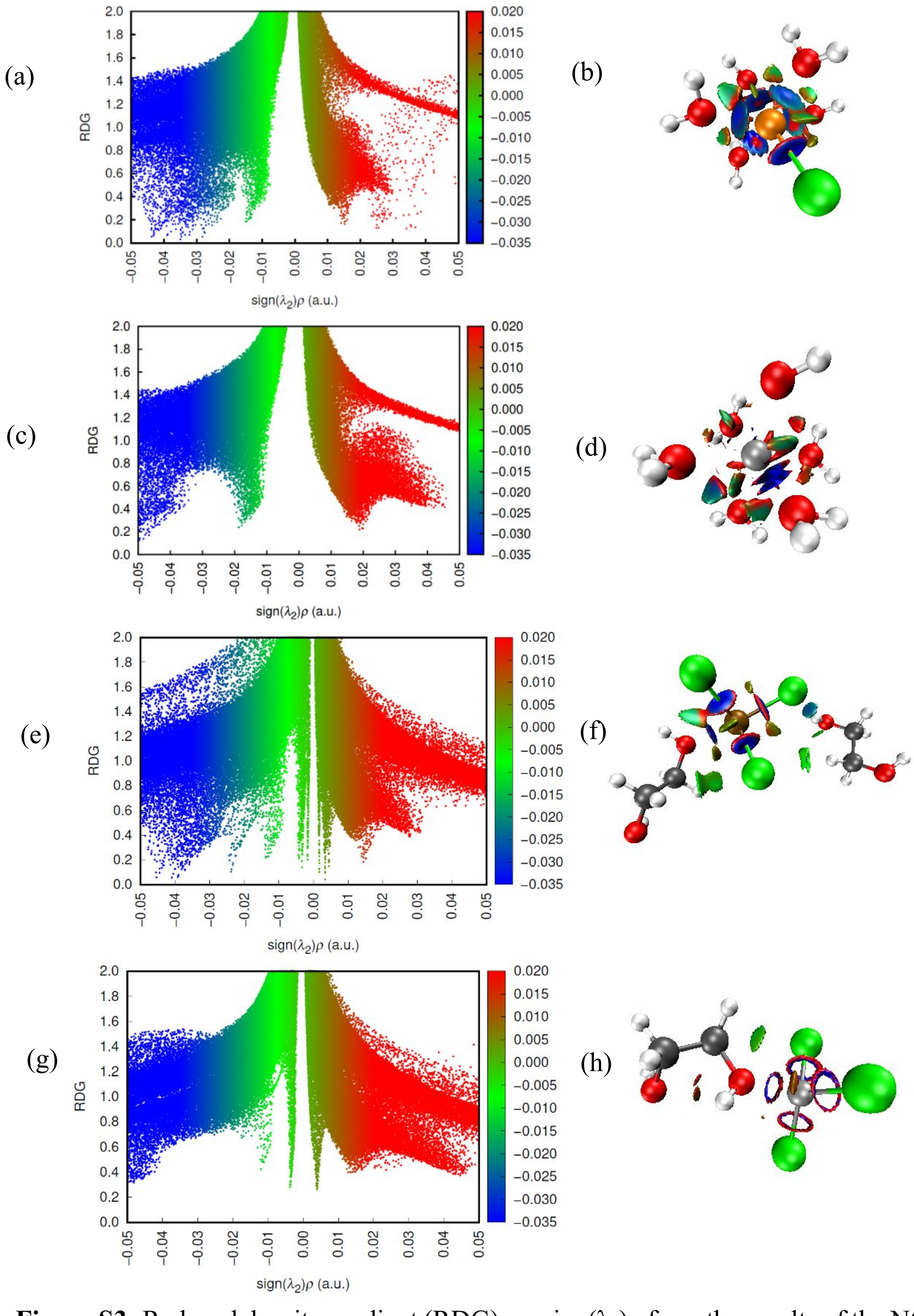

Figure S3: Reduced density gradient (RDG) vs. sign(λ_2)ρ from the results of the NCI and QTAIM analyses for Fe^{2+} ion in FeNiW5580 system (a) and intermolecular interactions for the species (b); Ni^{2+} ion in FeNiW5580 system (c) and intermolecular interactions for the species (d); Fe^{3+} ion in Fe3NiDES system (e) and intermolecular interactions for the species (f); Ni^{2+} ion in Fe3NiDES system (g) and intermolecular interactions for the species (h).
